# What’s the catch? Profiling the benefits and costs associated with marine protected areas and displaced fishing in the Scotia Sea

**DOI:** 10.1371/journal.pone.0237425

**Published:** 2020-08-12

**Authors:** Emily S. Klein, George M. Watters

**Affiliations:** 1 Antarctic Ecosystem Research Division, Southwest Fisheries Science Center, National Marine Fisheries Service, National Oceanic and Atmospheric Administration, La Jolla, CA, United States of America; 2 The Farallon Institute, Petaluma, CA, United States of America; University of Plymouth, UNITED KINGDOM

## Abstract

Both costs and benefits must be considered when implementing marine protected areas (MPAs), particularly those associated with fishing effort displaced by potential closures. The Southern Ocean offers a case study in understanding such tradeoffs, where MPAs are actively being discussed to achieve a range of protection and sustainable use objectives. Here, we evaluated the possible impacts of two MPA scenarios on the Antarctic krill (*Euphausia superba*) fishery and krill-dependent predators in the Scotia Sea, explicitly addressing the displacement of fishing from closed areas. For both scenarios, we employed a minimally realistic, spatially explicit ecosystem model and considered three alternative redistributions of displaced fishing. We projected both MPAs to provide positive outcomes for many krill-dependent predators, especially when closed areas included at least 50–75% of their foraging distributions. Further, differences between the scenarios suggest ways to improve seal and penguin protection in the Scotia Sea. MPA scenarios also projected increases in total fishery yields, but alongside risks of fishing in areas where relatively low krill densities could cause the fishery to suspend operations. The three alternatives for redistributing displaced fishing had little effect on benefits to predators, but did matter for the fishery, with greater differences in overall catch and risk of fishing in areas of low krill density when displaced fishing was redistributed evenly among the open areas. Collectively, results suggest a well-designed MPA in the Scotia Sea may protect krill-dependent predators, even with displaced fishing, and preclude further spatial management of the krill fishery outside the MPA. More broadly, outcomes denote the importance of delineating fishing and predator habitat, spatial scales, and the critical trade-offs inherent in MPA development.

## Introduction

Globally, fisheries managers and the scientific community increasingly recognize the value of shifting towards an ecosystem approach to fisheries management, with some advocating that marine protected areas (MPAs) be used to facilitate such a transition (e.g., [1]). MPAs can be useful, even essential, for reversing various anthropogenic impacts on marine resources and ecosystems (e.g., [2–6]). Protected areas may buffer against uncertainty [1, 7, 8]; enhance habitat protection, biodiversity, and population conservation (e.g. [9–11]); and increase the biomass, density, body size, and age distribution of species (e.g., [5, 6, 12–14]). MPAs may also boost fishery yields (e.g., [5, 11, 15–17]) and amplify the benefits of non-consumptive ecosystem services [18].

Despite these their advantages, MPAs can also present significant challenges. There may be social and economic consequences when MPAs deny stakeholders access to valuable, formerly available resources (e.g., [19–25]). Unanticipated ecological risks may also result from the establishment of an MPA, with redistributed human usage being a significant driver of such risks. For example, once displaced from MPAs, fishing effort may become more concentrated elsewhere, with consequent negative effects on commercially targeted and untargeted stocks, recovering and protected species, and habitats (e.g., [19, 26–29]). Collectively, adverse outcomes can undermine stakeholder buy-in and prevent an MPA from realizing desired policy and management objectives. Therefore, previous researchers have urged that MPA planners explicitly consider potential fishery outcomes, including fishing-effort displacement (e.g., [21, 22, 25]).

Ecosystem models can facilitate and improve MPA design [4, 30], particularly if human activities are represented as part of the ecosystem [18, 19] and the potential outcomes of fishery displacement are projected [30]. By comparing results with and without protected areas, ecosystem models can illuminate broad system implications that highlight benefits, drawbacks, and unanticipated outcomes [30]. It is critical such assessments include the social and economic effects of a projected MPA, and its impacts on the people whose activities will be affected. In particular, fishing-effort displacement may alter modeled outcomes [30, 31], but such effects are often overlooked or under-assessed [22, 23, 32, 33].

The Commission for the Conservation of Antarctic Marine Living Resources (CCAMLR or the Commission) is working to establish a network of MPAs in the Southern Ocean. CCAMLR is a pioneer in applying an ecosystem approach to marine conservation [34, 35], and spatial management is a core tenant of the convention establishing its management competency and authority. Recognizing that protected areas are useful for achieving its management objectives, the Commission established two MPAs: the South Orkney Islands Southern Shelf MPA and the Ross Sea region MPA [36]. Additional protected areas are under development in East Antarctica, the Weddell Sea, and a region including the Antarctic Peninsula and southern Scotia Sea (known as “Planning Domain 1”) [37].

CCAMLR considers both ecosystem and fishery needs when developing MPAs. Antarctic krill, *Euphausia superba* (hereafter krill), are a key forage species for numerous predators around the Antarctic Peninsula and in the southern Scotia Sea, including penguins, seals, whales, and fishes. An international krill fishery also operates in this region, and its spatio-temporal activity overlaps with that of the foraging predators [38, 39]. Since some predator populations may be sensitive to changes in krill availability [40–42], they may be vulnerable to risks from elevated competition for this common resource when and where fishing and foraging predators coincide [e.g. 43]. Given that such risks can be managed by redistributing fishery catches to reduce competition [44, 45], the Commission defined a set of “small-scale management units” (SSMUs, [46]) within statistical Subareas 48.1, 48.2, and 48.3 to facilitate the distribution of catches throughout the Scotia Sea. However, SSMU-specific catch limits have yet to be adopted. Establishing an MPA in Planning Domain 1 might augment CCAMLR’s management strategy in the Antarctic Peninsula and southern Scotia Sea region, if the socio-ecological risks and costs associated with displaced fishing can be evaluated and minimized.

To assess risks to this complex human and natural system, we evaluated and compared two MPA scenarios in Planning Domain 1. We focused on both the conservation of krill-dependent predators and the performance of the international krill fishery, and examined how scenario outcomes could be altered by different redistributions of the fishing effort displaced by an MPA. For this work, we employed an established ecosystem model that has been used to assess trade-offs between ecological conservation and human use. This model is dynamic, spatially-explicit, and minimally realistic (*sensu* [47]), although it does not include fleet dynamics and assumes both foraging and fishing are based on recent patterns from current data. Our objective was to profile the benefits and costs inherent in implementing an MPA within Planning Domain 1, and to provide insight to facilitate decision-making. In particular, we were interested in whether MPA design could effectively delineate predator and fishing distributions in a way that would benefit predator populations and also optimize outcomes for people. The value and aim of this work is not to provide specific predictions, but to better understand trade-offs relevant to protected areas in the Southern Ocean–thus it also supplies a case study for similar spatial management elsewhere.

## Methods

### The ecosystem model

**Fig 1:**
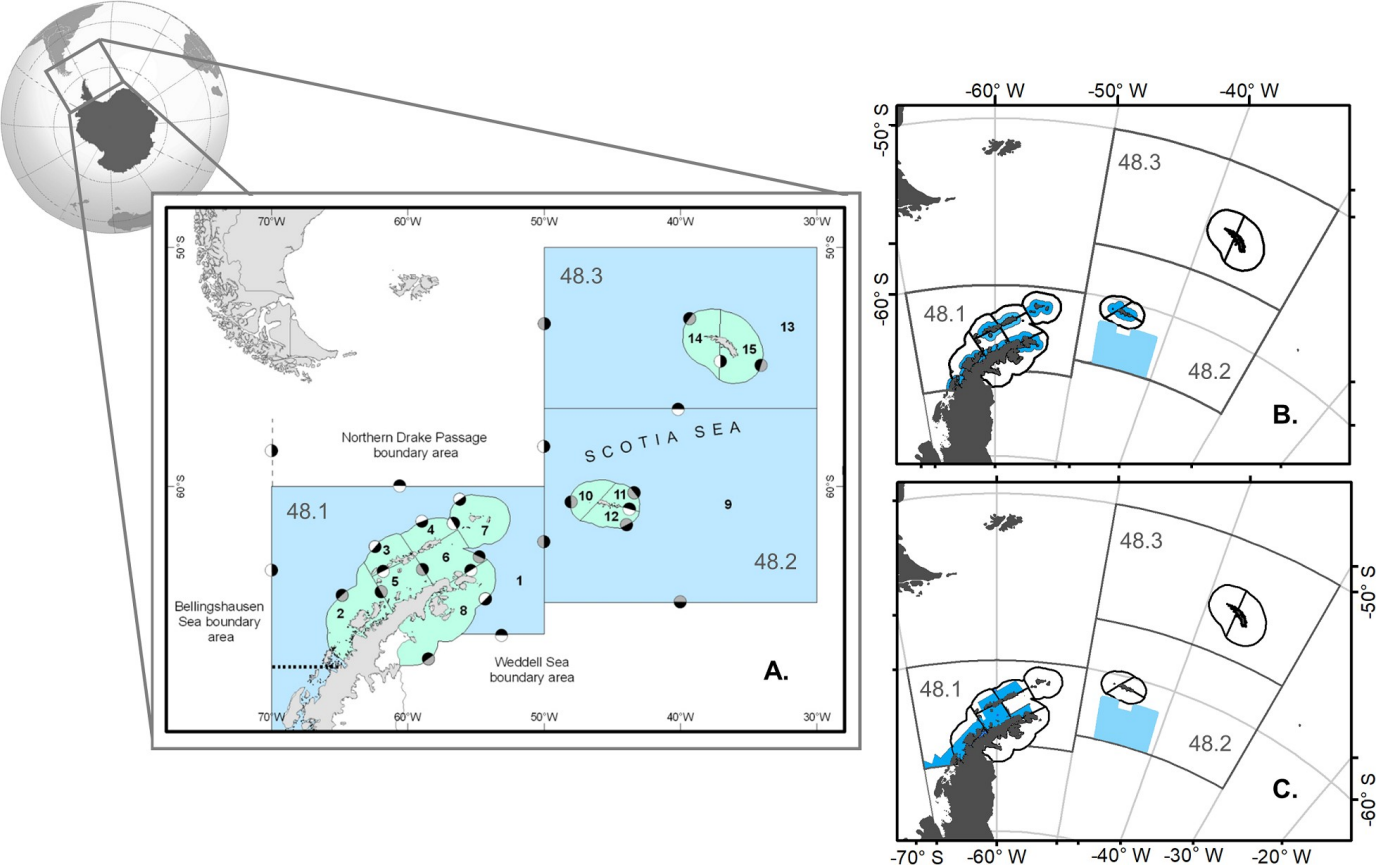
Spatial structure of the ecosystem model (A) and the two MPA scenarios (B-C). The ecosystem model arena (A) is comprised of 15 SSMUs (labeled in black) within three Subareas (labeled in grey) (Subarea 48.1 includes eight SSMUs, Subarea 48.2. four SSMUs, and Subarea 48.3 three SSMUs). The modeled MPA scenarios are simplified representations of (B) the initial MPA proposed to CCAMLR by the Delegations of Argentina and Chile (the D1MPA scenario) in 2017 [54], and (C) a scenario based on U.S. stakeholders’ spatial protection priorities (the US10 scenario). In (B-C), closed areas specific to each scenario are shaded in darker blue, and the existing South Orkney Islands Southern Shelf MPA is in lighter blue.

To project the consequences of each MPA scenario, we used a spatially explicit model of the Scotia Sea ecosystem written in the R language [48] and described by Watters et al. [45]. Hill and Matthews [49] provided sensitivity analysis, and much of the data used was vetted by CCAMLR and peer review [50]. This model is minimally realistic as opposed to representing as much of the system as possible––a “model of intermediate complexity for ecosystem assessments” (MICE, [47]) detailing a specific subset of a coupled system to explore trade-offs between krill-dependent predators and the krill fishery. To this end, the model focuses on krill, krill-dependent predators (multi-species groups of whales, penguins, seals, or fishes), and the fishery for krill. It includes two time-steps (seasons) per year, and spatially represents three subareas in the Scotia Sea divided into 15 coastal and offshore SSMUs. Twelve of the SSMUs in two of the subareas occur within Planning Domain 1 and include a potential MPA (Fig 1).

For the model ecosystem, krill-dependent predators breed in one SSMU and forage across wider areas composed of multiple SSMUs, with these foraging distributions informed by recent tracking data. Delay-difference equations describe the dynamics of each predator group and krill, and the post-larval biomass of krill in each SSMU is a function of stochastic recruitment and area-specific mortality and movement. Available krill biomass is estimated at the beginning of each time step, and competition arises when this biomass is insufficient to satisfy the combined demand of predators and the fishery. Four “reference parameterizations” facilitate the consideration of key uncertainties by bracketing plausible rates of krill movement between SSMUs (no movement and movement as passive drifters), and relationships between krill biomass and the effective numbers of breeding predators (hyperstable and linear) [45].

Our ecosystem model simulates management strategies that limit the overall catch taken by the krill fishery, both in space and seasonally. Currently, the catch limit for the krill fishery is set much lower than the total precautionary catch limit, and this will continue until such time as CCAMLR implements a spatial management strategy that successfully mitigates risks to krill predators [51]. Therefore, in theory, an effective approach to spatial management would allow CCAMLR to increase allowable catches from the current level to the total precautionary limit. As we are modeling MPAs as a potential spatial management approach, we chose to assume their implementation–especially given they prohibit fishing in some areas–would also see the fishery allowed to develop towards the total limit. We then used that total limit in our model instead of arbitrarily choosing a lower one. Therefore, the catch limit realized in the model is the product of (1) the initial krill biomass across the model arena and (2) the harvest rate used to set the overall catch limit for krill in this region (0.093). The spatio-temporal distribution of catches in the ecosystem model was determined by recent (2009–2017) fishing data ([52] and see [45]). Catches currently taken within the closed areas and thus displaced by the two MPA scenarios were redistributed to areas that remained open to fishing using three alternative redistributions of catch, explained as follows.

### Implementation of the marine protected areas

Here, we aim to provide feedback on a formally proposed MPA. Therefore, the first of the two MPA scenarios we considered represents the initial, formal MPA proposal submitted to CCAMLR by the delegations of Argentina and Chile in 2017 [53, 54 and discussed in 37 and 55] and referred to as the Domain 1 MPA, or “D1MPA” [55] (Fig 1). The second scenario derives from an assessment of spatial protection priorities held by a small group of U.S. stakeholders from government, academia, non-governmental organizations, and the tourism and fishing industries ([56], hereafter “US10”; Fig 1). The US10 scenario was presented to CCAMLR Members to catalyze thinking about spatial protection within Planning Domain 1 and inform them of some U.S. priorities for spatial protection. The US10 scenario is not a formal proposal, but, given its basis, we compared it with the D1MPA scenario to obtain information useful for improving the D1MPA, and we are not aware of other MPA scenarios designed to satisfy stakeholders in this region. Both modeled scenarios included the existing South Orkney Islands Southern Shelf MPA [57]. For simplicity, all areas within the boundaries of each MPA scenario were treated as “no-take” areas closed to fishing; conversely, areas outside these boundaries were considered open to fishing in our model. We note that the two MPAs are largely confined to the SSMUs along the coast; neither scenario includes substantive protection in pelagic, off-shore SSMUs beyond that included in the South Orkney Islands Southern Shelf MPA.

There is no protected area in the northern Scotia Sea (Subarea 48.3) in either scenario, as this lies outside Planning Domain 1 (i.e., Planning Domain 1 only includes Subareas 48.1 and 48.2). Subarea 48.3 is within the spatial arena of our ecosystem model (i.e., the model arena encompasses Subareas 48.1–48.3), as the fishery and predators utilize the entire area. Therefore, our model also provides an opportunity to test outcomes of an MPA in areas that are currently outside management units within the MPA, but are connected to such closed areas by the movement of krill, displaced fishing, and predator foraging.

MPAs may be most effective where they protect predator habitat, and we used available telemetry data to determine the areas where predator groups foraged in the model (except for fish, for which we had no telemetry data) (as in [38]). For this, a state-space model [58] was fitted to available telemetry data to account for uncertainty and estimate the proportion of time predators spent inside or outside the closed areas defined by each MPA scenario (see S1 File for details). We considered these proportions representative of the amount of krill a predator demands from inside an MPA (“foraging percentage”, Table 1) and compared this with modeled outcomes to determine if predator foraging inside an MPA–and therefore effectively delineated from possible competition with fishing–coincided with possible ecological benefits.

**Table 1:** SSMU-specific, seasonal percentages of the total krill a predator group demands for that season (i.e. foraging percentages) from areas closed to fishing in each of the two MPA scenarios.

SSMU	Penguins		Seals		Whales		Fish	
	*Summer*	*Winter*	*Summer*	*Winter*	*Summer*	*Winter*	*Summer*	*Winter*
**D1MPA**								
1					56.9%	36.8%	3.4%	3.4%
2	86.6%	55.7%					30.7%	30.7%
3	95.7%	35.9%	53.6%	8.5%			50.3%	50.3%
4	95.0%	27.8%	53.6%	8.5%			33.5%	33.5%
5	95.0%	28.7%					64.7%	64.7%
6	85.3%	53.6%					42.0%	42.0%
7	94.9%	27.0%	53.6%	8.5%			35.0%	35.0%
8	76.7%	59.3%					17.0%	17.0%
9					5.5%	0.0%	11.5%	11.5%
10	94.8%	8.0%					11.0%	11.0%
11	77.0%	3.3%					31.6%	31.6%
12	90.3%	7.3%					28.8%	28.8%
**US10**								
1					82.4%	56.7%	14.3%	14.3%
2	28.0%	46.1%					60.7%	60.7%
3	49.4%	31.5%	65.5%	10.9%			43.9%	43.9%
4	49.3%	22.9%	65.5%	10.9%			59.1%	59.1%
5	49.3%	23.9%					75.3%	75.3%
6	25.7%	43.2%					22.4%	22.4%
7	0.0%	22.0%	0.0%	10.9%			0.0%	0.0%
8	7.7%	42.7%					0.8%	0.8%
9					50.0%	0.0%	11.5%	11.5%
10	0.0%	9.4%					0.0%	0.0%
11	0.0%	0.1%					0.0%	0.0%
12	0.0%	7.3%					0.0%	0.0%

Summer is October through March in the model, and winter is April through September. Foraging percentages are reported for the SSMU in which the predator groups recruit in the model, but predators forage in multiple SSMUs across the model arena. Foraging percentages >50% are shaded blue (darker blue at >75%); those <25% are in red. Empty (white) cells denote that predator group is not modeled to recruit in that SSMU.

Because both MPA scenarios were developed independent from SSMUs, which have not been implemented by CCAMLR, they cut across SSMU boundaries. However, the model is parameterized based on SSMUs. This difference necessitated splitting each modeled SSMU into two parts, one part inside the MPA and another outside of it, and then revising the spatial parameterizations of the ecosystem model. To do this, we developed additional R code [48] to split the state variables and parameters of the original, SSMU-specific reference parameterizations. The first step in this process was to superimpose the D1MPA and US10 scenarios on maps of the SSMUs (i.e. those in Fig 1). Using the MPA boundaries, we defined two areas within each SSMU, one inside (closed to fishing) and one outside the MPA (open to fishing). We then decomposed spatially-dependent SSMU model parameters to obtain the reference parameterizations for the open and closed areas. These include: initial abundances of krill in each SSMU, maximum krill recruitments to each SSMU, instantaneous rates of krill movement between SSMUs, recent krill catches taken from each SSMU, and proportional distributions of predator foraging effort among SSMUs. Non-spatial parameters remained the same (see S1 File for a full list of parameters and their decomposed values). This process did not alter the primary structure or application (spatially explicit, ecosystem-based risk assessment) of our model, nor did basic decomposition alter basic model dynamics (S1 File).

Decomposing the parameters allowed us to evaluate the MPA scenarios, and established a hierarchy of four spatial scales to organize model results and redistribute displaced catches. From largest to smallest, the spatial scales considered here are (1) the full model arena comprising all three subareas considered in the model, (2) the subareas themselves (48.1, 48.2, 48.3), (3) the individual SSMUs, and (4) the open and closed areas in each SSMU created by implementing the MPA scenarios. However, the only scales of use in management are subareas, and the closed and open areas of the D1MPA are only the potentially implemented outcomes. Thus, the point of varying scale here is less about management in reality and more about exploring if outcomes change across spatial scales, which we have found to be significant in previous work [42].

### Redistribution of displaced catches

To explore how displaced catches might affect krill predators and the fishery in each MPA scenario, we maintained the overall catch limit and considered three alternatives for redistributing fisheries displaced from the closed areas. We estimated the catch displaced by the MPA closure using the overall catch limit and the average proportional distribution of catches during the 2009–2017 fishing seasons (see S1 File). For example, if the overall catch limit for an SSMU is 100 t and the fishery in that SSMU had taken 20% of its catch within the projected closed area, as defined by the scenario, up to 20 t of displaced catch was redistributed outside the MPA. The model sums catches displaced from all closed areas, then allocates the displaced catches across open areas at varying spatial scales. These three “redistribution alternatives" sum and then reallocate catches (1) across the statistical subarea in which catches originated (“Regional” redistribution alternative), (2) from the closed to the open areas of the SSMU in which the catches originated (“Local” alternative), or (3) across all open areas in the model arena in proportion to the average 2009–2017 catch distribution (“Current” alternative). Then we allocated these redistributions seasonally based on the seasonal distribution of recent catches (2009–2017 [52]). We note that redistributing catches based on average catch distributions recorded during 2009–2017 is probably inadequate for characterizing recent patterns in the fishery, which currently focuses on Subarea 48.2 during early summer, switches to Subarea 48.1 in late summer and early winter, and moves to Subareas 48.2 and 48.3 after the catch limit in Subarea 48.1 is achieved, but chose to rely on the published fishery data.

We based our redistribution alternatives on existing literature describing the behavior of fisheries following the establishment of closed areas. The Local alternative is akin to “fishing the line” (e.g., [24, 59, 60]), where vessels fish as close as possible to the boundaries of closed areas from which the displaced catches originated. The Current alternative assumes that fishers prefer recent fishing locations, perhaps over areas proximal to the closed areas (e.g., [61–63]), and wherein fishing vessels self-sort and redistribute to areas with the highest expected catch (e.g., [64, 65]). In contrast, the Regional alternative reflects additional regulation that redistributes fishing activities across open areas to minimize the ecosystem risks posed by unintended concentration of displaced catches [23, 63].

### Model implementation

We ran 1001 Monte Carlo trials (with random variations in krill recruitment) for each of the six combinations of MPA scenario, reference parameterization, and alternative redistribution of displaced catches. We also projected a “counterfactual” scenario [30], i.e. a “No MPA” reference scenario in which fishing was not displaced from the “closed” portion of an SSMU. All scenarios were initiated by conditioning on ecosystem dynamics from 1970–2007 [45], and the resulting estimated model abundances were then decomposed into portions inside and outside the MPAs. We then simulated a period of thirty years, with two seasons (summer and winter) per year, and averaged results across the parameterizations as in Watters et al. [45] and Klein et al. [42].

We computed total predator abundances within each SSMU in the last year of each simulation, focusing on penguins and seals to ensure results were manageable in the main text and as example species groups as these critical land-based predators have the potential for competition with the krill fishery (e.g. [38]). We also calculated two fishery-performance metrics. The first was total catch during the last year of each simulation. However, total catch may not fully capture important consequences for a fishery, i.e. the MPA may displace the fishery into open areas where krill are more difficult to catch (as noted in Hill et al. [66]). Therefore, our second fishery metric quantified the probability that the fishery would find itself in areas of low krill density during the model simulation. Like Watters et al. [45], we set a threshold for this density as 15 g∙m^-2^, and tallied how often season-specific krill densities fell below this threshold in areas open to fishing, which we termed “threshold violations”. We computed the average number of threshold violations per model run across the 1001 trials, and then the probability of such violations by dividing this average by the total number of seasons during the 30-year trial (i.e., 60 seasons).

The relevant code and model inputs are available online via https://github.com/EmilyKlein/KPFM2 and https://github.com/EmilyKlein/KPFM2_MPA_FBM.

## Results

**Fig 2:**
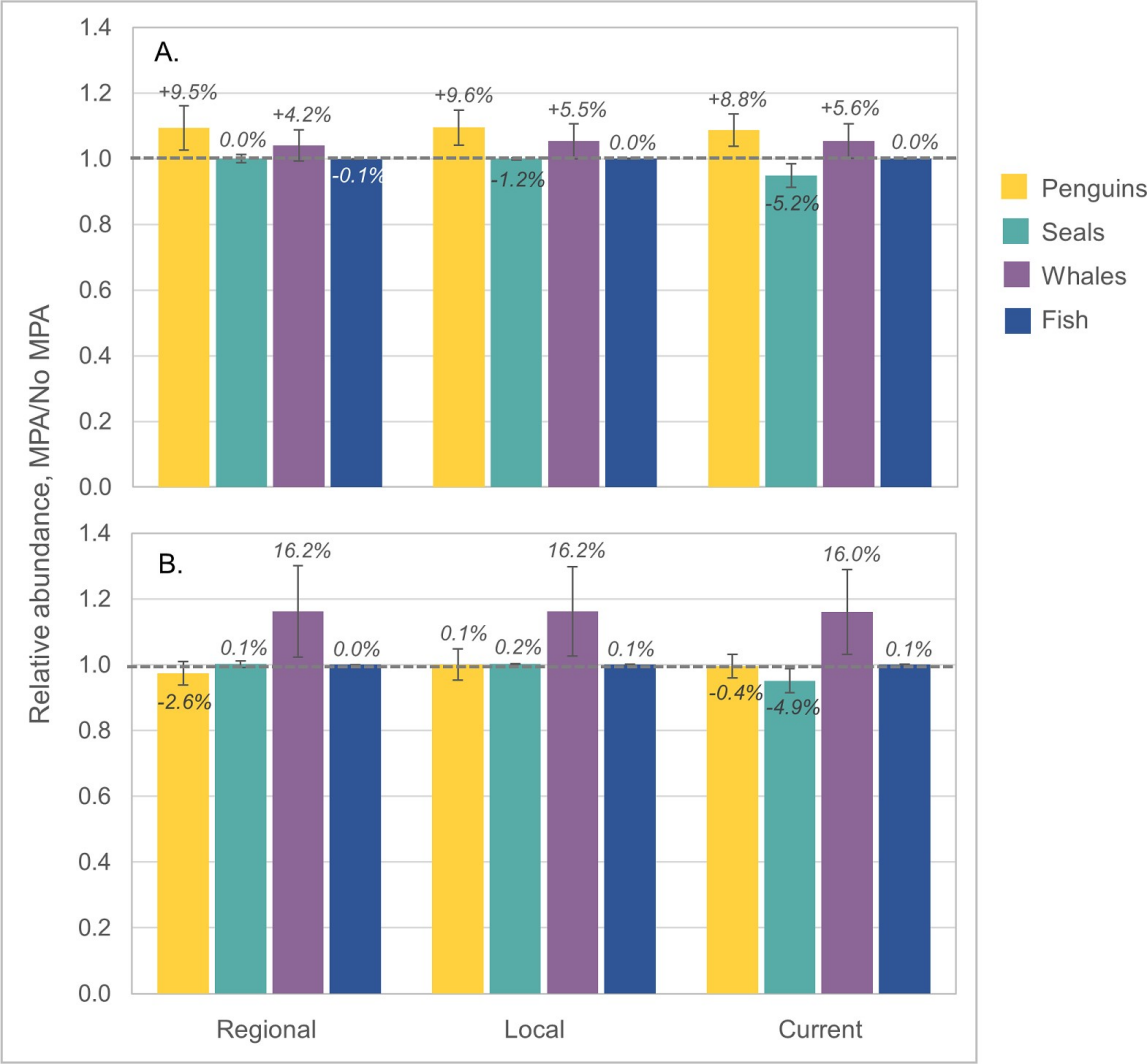
Relative changes, at the scale of the model arena, in overall predator abundance by species group and redistribution alternative given two MPA scenarios. Percent change in overall predator abundance by species group (penguins in yellow, seals in teal, whales in lavender, and fish in blue) during the final year of the model run for the (A) D1MPA and (B) US10 scenarios relative to the No MPA reference. The Regional redistribution alternative is the first set of bars, followed by the Local and Current alternatives. The dashed horizontal line at 1.0 indicates no change in modeled results between the MPA scenario and the No MPA reference. Bars above the line indicate increased abundance with the MPA, and bars below the line indicate population declines. Percent changes are labeled at the top of each bar, and error bars represent the standard deviation from the mean across all simulations in a scenario, capturing the variability across the four parameterizations.

Results were sensitive to the analytical spatial scale. At the scale of the entire model arena, we did not find major impacts on predator abundance in either of the MPA scenarios tested (Fig 2). Across redistribution alternatives, the model projected slight increases in total penguin (+9–10%) and whale (+4–6%) abundances under the D1MPA scenario, and larger increases with whales (+16%) under the US10 scenario. However, US10 showed a slight decline for penguins (-3%) under the Regional alternative, and seals declined in both MPAs under the Current alternative (-5%). All other combinations of MPA scenario and redistribution alternative indicated no change in abundance aggregated at the model arena scale, and the fish group was generally unaffected.

**Fig 3:**
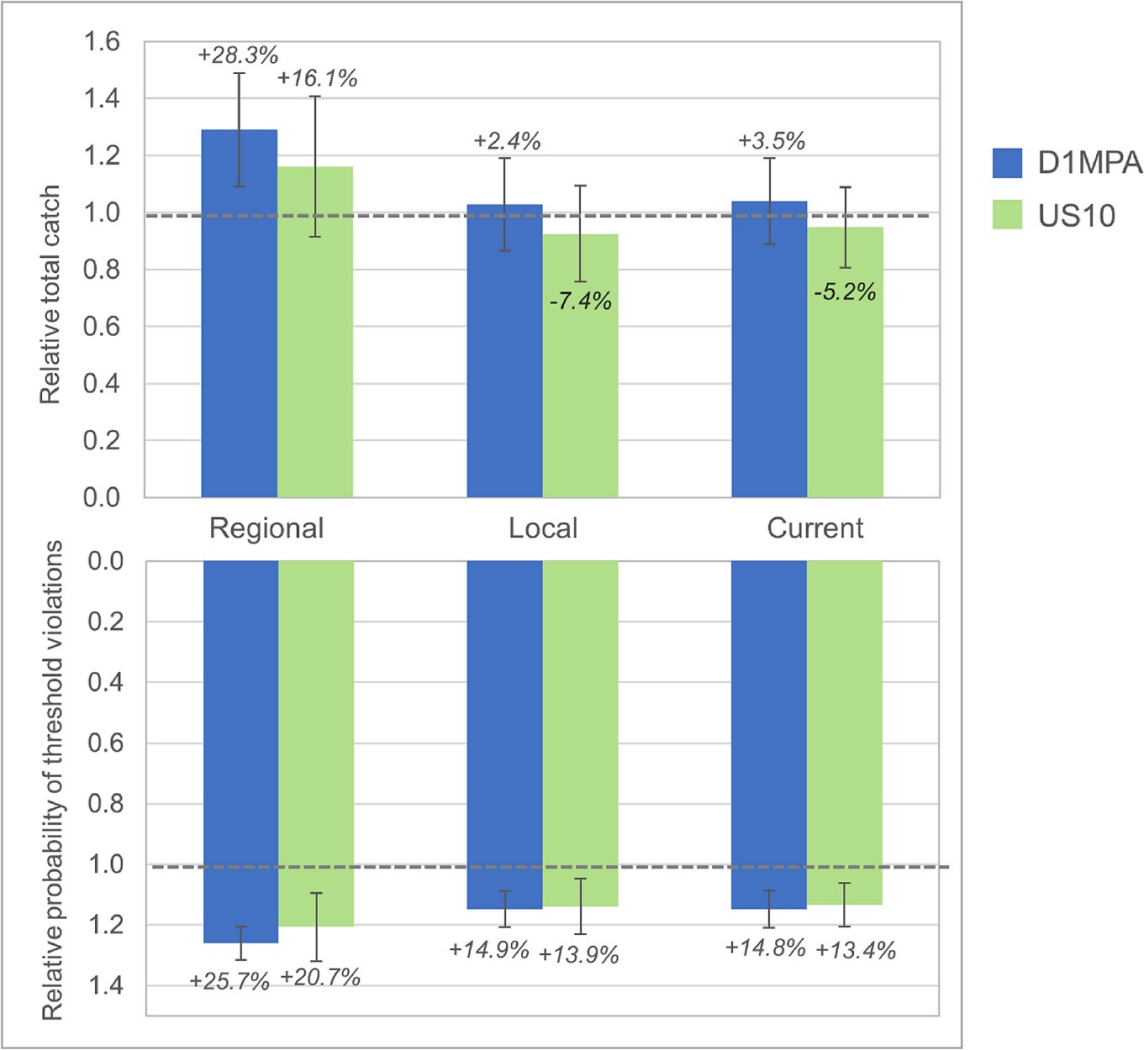
Relative total catches and probabilities of a threshold violation, at the scale of the model arena, across all redistribution alternatives in both MPA scenarios. Relative total krill catch in the final year of the model run (top bars) and the average probability of a threshold violation (bottom bars) by redistribution alternative for each MPA scenario (D1MPA in blue, US10 in green) relative to the No MPA reference. All other details as in Fig 2.

At the scale of the model arena, the krill fishery experienced relatively greater effects (Fig 3). Under the Regional alternative, both MPA scenarios yielded larger catches (+28% for D1MPA, +16% for US10). Catches under the Local or Current alternatives increased only slightly for the D1MPA (+2 and +4%, respectively) and declined for US10 (-7% and -5%). The probability of threshold violations (fishing in areas of low krill density) increased for both MPA scenarios and all three redistribution alternatives. Under the Local and Current alternatives this probability increased 13% to 15% in both MPA scenarios, whereas under the Regional alternative the probability increased even more: +26% for D1MPA and +21% for US10.

**Fig 4:**
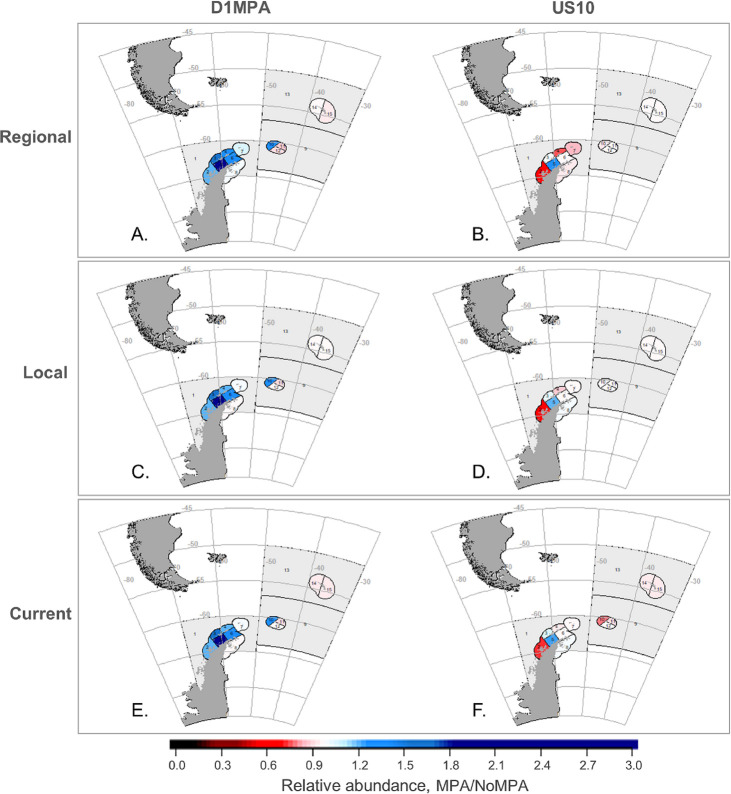
Outcomes for penguins by SSMU in both MPA scenarios. Changes in penguin abundance with an MPA relative to the No MPA reference (i.e. MPA/No MPA), with the top row (A and B) the Regional redistribution of displaced catches, the middle row (C, D) the Local alternative, and bottom row (E, F) the Current alternative. The left column illustrates results from the D1MPA scenario (A, C, E) and the right from US10 (B, D, F). Grey indicates areas where the species group is not modeled to recruit. Note that changes are relative to the no MPA scenario within each SSMU, not to overall change.

**Fig 5:**
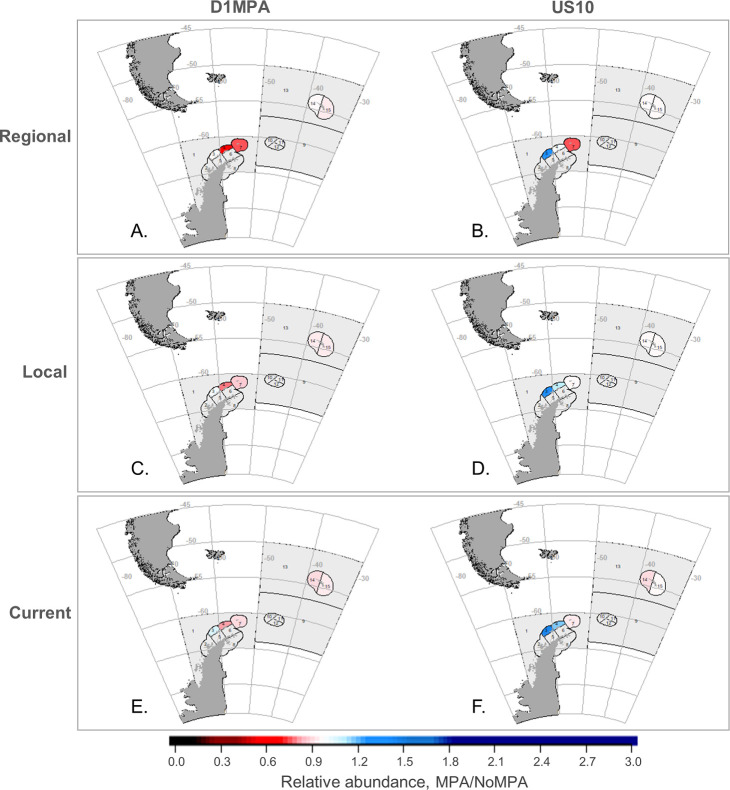
Outcomes for seals by SSMU in both MPA scenarios. Change in seal abundance with the MPA relative to the No MPA scenario (i.e. MPA/No MPA); all other details as in Fig 4.

**Fig 6:**
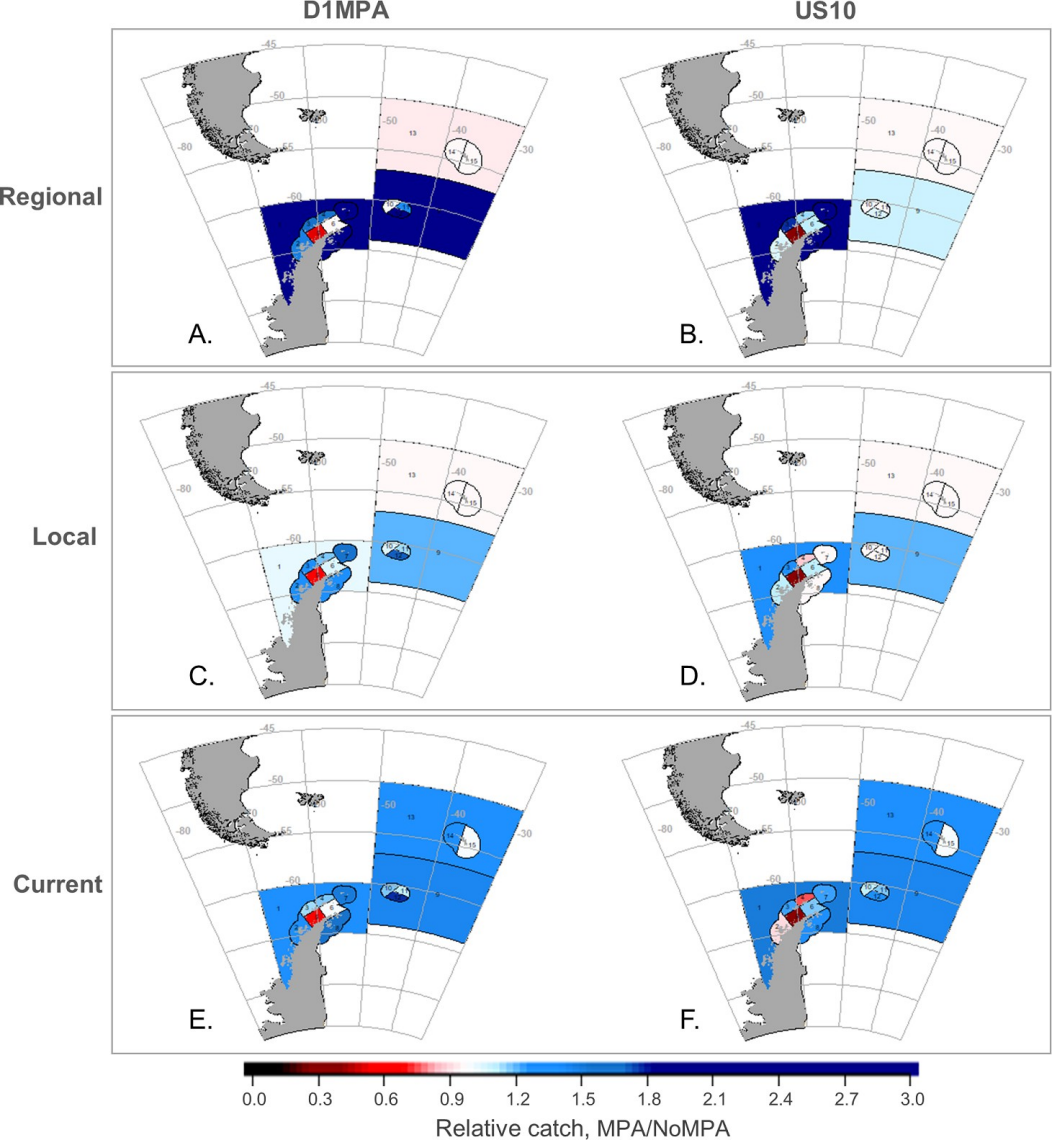
Outcomes for the fishery by SSMU in both MPA scenarios. Change in catch with the MPA relative to the No MPA scenario (i.e. MPA/No MPA); all details as in Fig 4.

As in Klein et al. [42], results aggregated across the model arena masked greater differences at smaller spatial scales of analysis (Figs 4–6). At the SSMU scale, the important takeaways are overall patterns of model projections, and, to keep figures in the main text manageable, we report results here for the penguin and seal groups, land-based predators most likely to overlap with the krill fishery (e.g. [38]). Results for whales and fish are provided in S1 File. For penguins, the D1MPA scenario yielded increasing populations in a greater number of SSMUs, with only a few minor local declines (Fig 4). Like the outcomes at the model-arena scale (Fig 2), penguin responses to the D1MPA were similar across the three redistribution alternatives at the SSMU scale, while the US10 scenario showed a wider range of responses. For seals, however, outcomes at the SSMU scale revealed more substantial differences both by MPA and redistribution alternative (Fig 5), although there was little change in the abundances of seals in both MPA scenarios at the scale of the model arena (Fig 2). Outcomes also differed across MPA scenario and redistribution alternative for fishes, and whales experienced slight increases or little change in the SSMUs where they were modeled (S1 File).

Spatial patterns also emerged in terms of fishery catches (Fig 6). Overall, outcomes at the SSMU-scale were somewhat more dependent on redistribution alternative than on MPA scenario. The fishery saw a range of catch increases, with few declines. The strongest decline in catch consistently appeared in the southwestern Bransfield Strait (SSMU 5). As overall patterns showed increased catches in the other SSMUs, declines in SSMU 5 may have been a strong driver of arena-wide catch declines (Fig 3). This is due in part to the reallocation of catch displaced by either MPA from this SSMU, but changes in catch were not entirely explained by differences between the MPA scenarios and the No MPA reference (see S1 File). Finally, we note threshold violations were more likely for many SSMUs in both MPA scenarios, in agreement with results at the scale of the model arena (Fig 3), although threshold violations within the pelagic SSMUs––those with some of the highest potential for low krill densities––were similar between the two MPA scenarios (S1 File).

**Fig 7:**
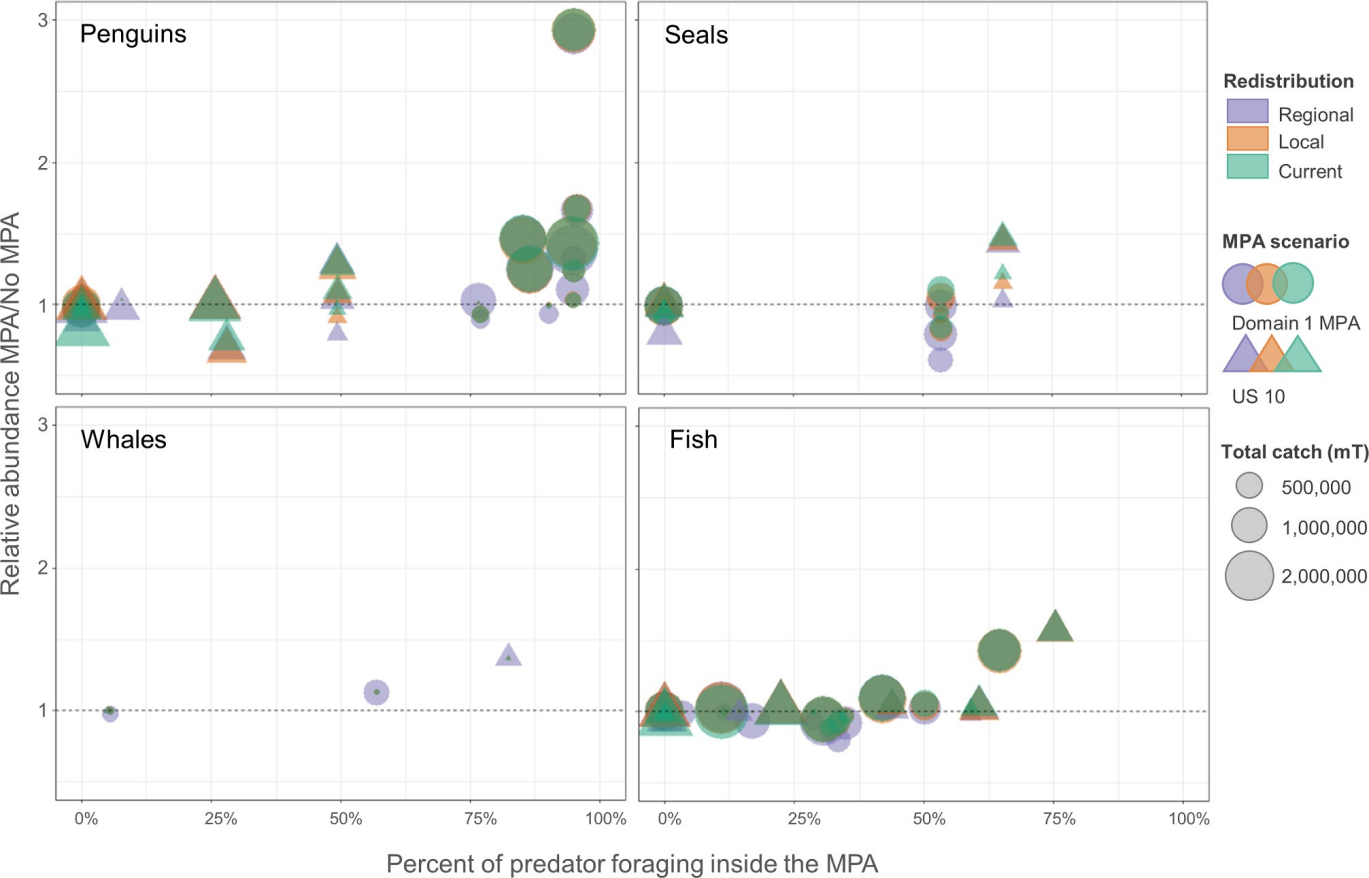
Relative abundances of species groups by SSMU given percentages of foraging inside an MPA. Relationship between the percent of foraging during summer (the breeding season) that occurs inside an MPA (x-axis) and change in abundance of each species group (penguins, seals, whales, and fish) relative to the No MPA reference (MPA/No MPA, y-axis). Each point represents an SSMU: circles are the D1MPA scenario and triangles US10. Colors denote redistribution alternative (Regional is purple, Local orange, and Current green). The dashed line at y = 1.0 indicates where there is no change in abundance with an MPA. Relative sizes of the points indicate the amount of catch in that SSMU; note many points overlap, indicating no difference across the redistribution alternatives for catches displaced by an MPA.

We also considered whether increases in predator abundance coincided with greater foraging in areas closed to fishing within MPAs, and if this could help explain our results. That is, we asked whether predator populations increase more often when locations where predators forage are closed to the krill fishery. To answer this question, we compared the percentage of predator foraging that occurred within closed areas in each MPA (Table 1) to the changes in relative abundance of predator populations (i.e., abundance in the final year of the MPA scenario relative to abundance in the final year of the No MPA reference). Generally, our results showed that abundance of all predator groups was likely to be higher when foraging occurred inside an MPA during the summer breeding season (Fig 7; we did not report patterns for the winter season as many predators depart to forage outside the model arena [45]). For most of the groups, differences between the MPA scenarios depended upon which scenario protected the largest foraging area. For penguins, the D1MPA (circles in Fig 7) saw greater increases than US10 (triangles in Fig 7) since it protected a greater proportion of the birds’ foraging areas. The opposite was true for seals and whales, whose foraging distributions were more protected foraging under US10 than the D1MPA. For seals, a small increase in foraging protection in US10 over the D1MPA in some SSMUs (Table 1) still returned a greater seal abundance. In contrast, while relative fish abundance also increased with an increase in protected foraging areas, no difference appeared between the two MPA scenarios. Notably, many increases in predator abundance were unaffected by how fishing displaced by the MPA was redistributed (as indicated by points of different colors fully overlapping one another in Fig 7).

Results further denote little connection between the amount of krill caught and relative changes in predator abundance (Fig 7). We find no discernable relationship between symbol sizes in Fig 7, indicating the amount of krill caught in the open area within each SSMU, and the abundance of predators. These results also show: (1) large catch quantities only affected outcomes for predators if the krill were caught in areas where they forage, and (2) an MPA that effectively promoted increases in predator populations often did so alongside high krill catches outside protected areas.

Importantly, our results also revealed some declines in predator populations with an MPA in place (Figs 2, 4, 5 and 7). This counterintuitive finding suggests that displaced fishing may indeed increase risks for krill-dependent predators, but such declines more likely occur where an MPA does not sufficiently encompass the foraging habitat of predators (points below the y = 1 line in Fig 7). Indeed, as long as >50% of predator foraging areas occurred within closed areas, we found most predators did not decline in abundance. As the percentage of foraging habitat rose to and beyond 75%, all four predator groups approached their greatest abundance.

## Discussion

Why design an MPA that maximizes ecosystem benefits without considering social costs? This rhetorical question highlights the fallacy of assuming one simply needs to close most areas where predators and a fishery compete for a common resource. A challenging but more realistic task is to design an MPA that simultaneously manages human costs and ecosystem risks, such as from the potential adverse effects of displaced fishing. In our case in the Scotia Sea and Antarctic Peninsula, these costs and risks revolve around a fishery and predator populations all dependent on the same forage species, krill. These findings also advance evaluation methods for MPAs in general; profiling “bad” outcomes provides knowledge useful in developing “better” MPAs.

### Insight for MPA development in the Scotia Sea

#### Improving outcomes for the D1MPA

Our results hold particular insight for improving the D1MPA. Several authors have discussed the various tradeoffs inherent in krill-fishery management and for predators in the Antarctic (e.g., [43–45, 67]). These discussions relate to the spatial distribution of fishing, and tradeoffs typically contrast maintaining robust or resilient populations of krill-dependent predators with increasing potential krill-fishery yields. Because discussions about MPAs in Planning Domain 1 fit squarely in this setting, our projections of the potential ecological and social benefits and costs of implementing protected areas with different patterns of fishing displacement aim to advance CCAMLR’s work on MPAs.

Comparing results from the D1MPA and US10 scenarios is also useful for revising the D1MPA proposal [53, 54] to increase benefits while considering costs for the fishery, the ecosystem, and the potential consequences of fishing displaced by protected areas. Active debate on the D1MPA proposal includes dialogue on closed areas delimited by a 30-km buffer around the Antarctic Peninsula, South Shetland Islands, and South Orkney Islands coastlines (Fig 1B) [53]. This buffer is particularly relevant to the protection of Antarctic fur seals (*Arctocephalus gazella*) and three penguin species, which must provision their offspring in breeding colonies on land, and are central place foragers during the austral summer. Given its origins for denoting priorities among U.S. stakeholders [56], inclusion of the US10 scenario can help consider additional stakeholder aspirations, and, as it provides further information, may aid in achieving consensus for an MPA in Domain 1, which is currently lacking [37].

Our results indicate that, within the D1MPA scenario we modeled, a 30-km buffer may fail to mitigate the risk of depleting Antarctic fur seals due to displaced fishing. Our model findings projected fur seals to fare better under the US10 scenario, likely because it encompassed more seal habitat in the southern Drake Passage than the D1MPA scenario (SSMUs 3 and 4). About 70–80% of Antarctic fur seal pups in the South Shetland Islands are born in SSMU 3 (M. Goebel, personal communication), and fur seals breeding in SSMU 3 spend significant amounts of time in waters where the bottom depth is shallower than 2000 m (see Fig 7 in [38]). The 2000-m isobath is about 40–60 km from the coastline in SSMU 3, so extending the buffer to 60 km may reduce the risks that D1MPA will exacerbate seal depletion.

Earlier work has shown the importance of more actively managing displaced fishing to offset detrimental economic and ecological impacts (e.g. [18, 27, 62]). Watters et al. [45] have argued the importance of spatially redistributing harvesting effort, in particular shifting catches offshore from coastal SSMUs to mitigate the ecosystem risks posed by krill fishing, but our conclusions with different MPA scenarios were less straightforward. We found that, if predator foraging is protected by an MPA, changes in population abundance can be insensitive to the redistribution of displaced catches, whether to neighboring areas, previously fished areas, or throughout the relevant subarea (Figs 4A, 4C, 4E and 7). However, the fishery itself can be impacted by the way displaced fishing is redistributed. We projected increased catches if fishing was distributed more regionally, but this benefit came with the increased costs of fishing in areas of low krill density (Fig 3 and S1 File). For these reasons, we suspect that, although broad redistribution of displaced fishing could mitigate novel risks from MPAs by avoiding the concentration of effort in open areas, the utility of doing so is case-specific and dependent upon the design of candidate MPAs. The fact that our Regional redistribution alternative increased the costs of fishing (Fig 3) but was not considerably more beneficial to predators (Figs 2, 4 and 5) suggests that CCAMLR can probably permit fishing vessels to self-sort among open areas if the D1MPA, or an improved version of it, is eventually established.

In 2018, Argentina and Chile used the preliminary results of our work to update their official proposal for an MPA in Planning Domain 1 [37, 68]. Their alacrity demonstrates the urgent need for scientific advice and the significant value of modeling the potential outcomes of protected areas in support of policy-making. Further, such urgency displays the critical need for understanding both the ecological benefits of proposed MPAs and their potential adverse consequences, especially regarding displaced fishing. We intend for our results to support continued dialogue on protected areas in the Southern Ocean, and our work is ongoing.

#### Further management implications for CCAMLR

Rationalizing MPAs with other tools of fishery management is an active topic of discussion (e.g., [23, 69]). We envision harmonizing customary krill-fishery management with MPAs by plugging the latter into the former. CCAMLR has a two-pronged strategy for managing the krill fishery: limiting (1) overall catch––primarily to conserve the krill stock, and (2) the spatial distribution of catches––primarily to conserve krill-dependent predators. If baseline risks posed by the overall catch limit and the current catch distribution are acceptable, and an MPA (with its accompanying displacement) does not exacerbate these risks for krill predators, that MPA might effectively substitute as the second element of CCAMLR’s management strategy. We argue our results indicate that such a substitution is feasible because our projected implementation of the D1MPA scenario exhibited demonstrable benefits for some predator populations, especially penguins (Fig 4). For seals, comparing results from both scenarios suggested revisions to the D1MPA that may reduce the potential for declines, which appeared in model results, namely, by more effective protection of seal-foraging areas. We also showed that additional limitations on the spatial distribution of fishing outside the D1MPA may be unnecessary if predator foraging is effectively protected (e.g. Figs 4 and 7). Thus, in our opinion, and given that the present goal of CCAMLR’s spatially explicit catch limits is to manage risks to predator populations, a well-designed MPA can potentially maintain acceptable risks to krill-dependent predators and substitute for other ways of spatially distributing krill catches (e.g., substitute for the spatial distributions considered by Watters et al. [45]). Of course, there are reasons to impose spatial catch limits other than the protection of krill-dependent predators, and should CCAMLR adopt a substantially different MPA in Planning Domain 1, further assessment may be required.

If CCAMLR decides to implement a suitably revised version of the D1MPA scenario and integrate the MPA with its management strategy for the krill fishery, the Commission could achieve multiple objectives simultaneously. The Delegations of Argentina and Chile proposed an MPA that aims to accomplish a broad suite of protection and conservation objectives [37, 53, 54, 68]. These objectives extend beyond conservation of krill-dependent predators, though some are linked to krill fishing. For example, the D1MPA aims to protect other species of krill and juvenile finfishes that are bycaught in the krill fishery. Other objectives of the D1MPA do not clearly link to krill fishing but aim to protect animals and habitats that occur in areas where the fishery operates, e.g., benthic communities. Thus, the closed areas comprising the D1MPA scenario encompass more than the foraging habitats of krill-dependent predators [53, 54]. Our model cannot test whether all of the objectives for the D1MPA are achievable, but CCAMLR’s Scientific Committee recognizes that “priority areas for conservation” occurring within the proposed MPA boundaries are justified by the available data [70]. Our model can test whether displacing krill fishing to achieve objectives unrelated to krill-dependent predators jeopardizes the achievement of related objectives. If CCAMLR adopts revisions to the D1MPA as proposed by the Delegations of Argentina and Chile in 2018 [68] to reduce the effects of displaced fishing on seals in the southwestern Drake Passage and penguins near the western South Orkney Islands, we expect the protected area can indeed satisfy a broad range of objectives. The potential utility of the D1MPA for achieving the various conservation objectives identified by Argentina and Chile is further supported by Dahood et al. [71], a comparative modeling study.

#### Insight for MPA development more broadly

Our modeled outcomes have implications for CCAMLR and the Southern Ocean, but what we learned provides insight for MPA development broadly as well. First, our results indicate that MPA design may significantly impact ecological outcomes, in our case, for krill-dependent predators. If a management objective is to protect predator populations, then an effective MPA can ensure predator abundance is largely unaffected by fishing displaced outside the MPA, and minimally impacted by large catches of the common prey resource. Our work indicates this effectiveness is achieved when at least 50–75% of predator foraging areas are closed to fishing within an MPA (Fig 7). When this level of protection is achieved, predators are buffered against decisions made about the distribution of fishing activity in open areas. Equally important, if an MPA does not sufficiently protect important foraging areas, redistributed fishing can increase competition in open areas where predators continue to forage, potentially causing their populations to decline.

Our findings also indicate that benefits to predators do not necessarily come at the cost of adverse effects on the fishery. High catches are possible even as predator populations increase when an MPA displaces fishing from foraging areas (Figs 3, 6 and 7). This outcome suggests that effective protected areas can be designed to result in weaker trade-offs for people. In fact, the redistribution of displaced fishing to specific locations may permit greater resource use by both fishers and predators (Figs 3, 6 and S1 File).

Potential catches may nevertheless depend on how fisheries respond to closed areas. Total catches may increase if displaced effort is widely redistributed as in our modeled Regional redistribution alternative, as opposed to redistribution locally or to areas where fishing effort is already concentrated (Fig 3). However, this effect may differ at smaller spatial scales (Fig 6) and be skewed by displacement from areas with high current effort (e.g. SSMU 5 in Fig 6 and S1 File). Further, projected increased catch may not manifest in reality if the fishery is shifted away from its current or preferred grounds to potentially less desirable areas.

Greater catches outside MPAs may come with other costs. We found that the overall probability of fishing in economically less desirable areas (i.e., threshold violations) increased in all MPA scenarios (Fig 3). As we did not model fleet dynamics, a threshold violation should be interpreted as the need for fishing vessels to move on due to low krill densities. This outcome is not surprising, given that an MPA in our model forces fishing to concentrate in smaller areas within an SSMU; this condition could increase the potential to deplete local krill resources and increase the probability of threshold violations. Moreover and to varying degrees, all redistribution alternatives increased catches in pelagic areas where krill densities are lower and the potential for threshold violations are higher (Fig 6 and S1 File). Regardless, this outcome underscores the need to balance improved yields against the depletion of local krill stocks, and the resulting potential for the fleet to move to or focus on other grounds, which can incur additional costs not modeled here (e.g. fuel, time, safety at sea). Thus, our work exemplifies the fact that possible consequences of protected areas for people are complex and extend beyond total catch alone.

Finally, differences between results aggregated over the entire modeled arena (Figs 2 and 3) and those at the SSMU scale (Figs 4–6) indicate the importance of assessing outcomes in a spatially explicit way. Outcomes at the scale of the model arena effectively masked important impacts of the MPAs at smaller spatial scales. This is especially pertinent given the potential for overlap with human use [38, 39] and the consequences of climate change [42] at finer spatial scales. Overall changes in species abundance may not signal local declines in areas more vulnerable to the effects of climate change [42]. Increases in total catch may also obscure spatial changes in fishing that could have substantial consequences for the fishery, e.g., because of safety risks, fuel and travel costs, or by forcing vessels onto less desirable fishing grounds due to the quantity or quality of the target species. The important point for management is to consider exploring when and if outcomes change with spatial scale, and to use that to address whether current schemes are appropriate. Here, we show there is potential for the current spatial scale of Subareas to mask outcomes and, possibly, important costs to the ecosystem and the fishery.

All of these outcomes provide specific guidance for MPA implementation aimed at conserving krill-dependent predators while maintaining a productive fishery in the Southern Ocean. They also hold insight for design of MPAs with similar goals more broadly. While delineating human use and species foraging on a common food resource may be intuitive for MPA success, here we highlight this importance. However, we also show that the potential negative consequences of increased effort from displaced human use can be avoided and that MPAs can supply benefits to people and predators, but that these outcomes will depend on how people respond to management action and may need to be balanced against other trade-offs and costs for resource users.

### Important caveats to our work

Most modeling comes with particular caveats. Previous studies (e.g., [42, 44, 45, 72]) describe many of the caveats in our work, including the representation of predators as aggregate groups, and the possibility that alternative functional relationships might better describe ecosystem dynamics in the Scotia Sea. Here, we add to the list of caveats that should be considered. To decompose our model’s reference parameterizations, we assumed random distributions of catches and krill densities throughout each SSMU (S1 File). The outcomes of our model may differ if catches and krill were not randomly distributed within SSMUs. For example, larger catches in a potential closed area where krill density is high imply greater fishery displacement, with consequently higher costs in open areas and lower risks for krill-dependent predators continuing to forage in the closed area. Indeed, catches and krill are heterogeneous in space, and substantial interannual variations have been documented in locations with the highest krill catches and densities (e.g., [66, 73], respectively). Dealing with such variability is important, but also implies a level of realism that is inconsistent with our simple hypotheses about krill movement and aggregated predator groups; that is, realism that is inconsistent with the current reference parameterizations of our model. In addition, we found the overlap of fishing and foraging to be critical, but these conclusions, as with any from models, are based on the reliability and accuracy of the data used. Fishery data published by CCAMLR is considered reliable, our other inputs and model structure have been vetted by previous peer review (e.g. [49, 50]), and here updates from tracking are based on recent surveys, but certainly new data may impact modeled results. Similarly, a final caveat is that the parameterizations used herein could be updated by re-tuning the model to data that have become available since publication of Watters et al. [45].

## Conclusion

When implementing an MPA like the D1MPA, tradeoffs must be considered to manage the costs of displaced fishing and mitigate the risks to predators that depend on the resource targeted by the fishery. Our results show that simply adjusting the size of closed areas to better encompass predator foraging may slow or halt the depletion of krill-dependent predators no matter how fishing effort redistributes in space. Our projections further indicate that these outcomes would have little impact on fishery catches, however the required shifts in fishing area may prove costly for people if vessels are displaced to less desirable locations where search costs may be higher, fishing conditions more dangerous, and krill quality is less desirable or inferior. We were unable to assess all of these fishery tradeoffs based on our model criteria. Yet they and other social tradeoffs form the crux of many broad debates about MPAs (e.g., [18, 19, 31]), which are best resolved in participatory processes that consider a range of perspectives and views [74, 75]. We previously asserted that it is fallacious to design an MPA to minimize the ecosystem effects of displaced fishing without assessing the social costs of closing critical fishing grounds. It is also fallacious to design MPAs that minimize displacement costs (i.e. by closing very small areas) or exacerbate both ecosystem effects and fishery costs (i.e. by closing previously unfished areas). In the Scotia Sea, such solutions are potentially pathological. Furthermore, they fail to recognize legitimate alternative perspectives and views held by various CCAMLR Members.

Fortunately, CCAMLR’s process for establishing MPAs is participatory. There are many opportunities to assimilate alternative perspectives into the design of the D1MPA. The Delegations of Argentina and Chile developed their initial (submitted in 2017) and revised (submitted in 2018) proposals over several years of consultation and collaboration with many colleagues, stakeholders, and other national delegations [37]. These consultations and collaborations continue as the other delegations evaluate the proposal and provide feedback to its proponents. Both the political decision to establish an MPA and its ultimate effectiveness often hinge on stakeholders’ acceptance of the tradeoffs that emerge during its design and implementation (e.g., [75] and references therein). Agardy et al. [74] asserted that “progressive MPA planning not only focuses on ecological design but also how protected areas will affect environmental and social outcomes,” but concluded that very few studies consider the detrimental ecosystem effects and the costs of displaced fishing. We hope to buck this trend. In combination, the efforts undertaken by the Delegations of Argentina and Chile to develop, propose, and revise the D1MPA, by other delegations that continue to review and comment on the large volume of research underpinning the proposal, and by our models that project the potential ramifications of the proposal, exemplify best practices in MPA planning (also [37]).

Collectively, our work and its connection to the CCAMLR process provide a case study that can guide MPA development elsewhere. Our results provide insight for decision processes that must balance ecological and human needs, particularly when both rely on the same or similar resources or in “wasp-waist” ecosystems [76]. This is especially useful given the real concern of increasing effort via the displacement of fishing from closed areas–and we show that this can undermine MPA goals of protecting species if that increased effort overlaps with important habitat and foraging grounds, thus aggravating competition instead of alleviating it. However, we also show that successful MPAs can provide conservation and human benefits, although the benefits for people need to be considered carefully. Finally, our work for CCAMLR also emphasizes the value in assessing more than one MPA scenario to help improve proposed designs, and the way in which work such as ours can fit into existing management structures.

## Supporting information

S1 File (DOCX)

S1 Table Update of area parameter, *A*_*i*_, in the ecosystem model. Spatial unit areas (m^2^) used in the original SSMU ecosystem model, with areas inside and outside the MPA calculated in the decomposed model for the D1MPA and US10 scenarios.
(DOCX)

S2 Table Model parameters and state variables. Description of the parameters in our ecosystem model and whether the parameter or variable was adjusted in the decomposition process (white rows) (adopted from Tables B2 and B3, Watters et al. 2013).
(DOCX)

S3 Table Additional updated input for krill and krill-dependent predators in the decomposed model versus the original. For krill, the initial density was multiplied by the proportion of area in either the MPA or no MPA portions: “inside MPA” and “outside MPA” columns sum to the “Original model” column.
(DOCX)

S4 Table Proportional distributions of krill catch for each of the 15 SSMUs. Proportions used in earlier versions of the model are under “Previous Model” (Table S1 in [5]). The updates used here under “Current Model” are derived from catches taken during the 2009–2016 fishing seasons and under limits at finer spatial scales specified by current management [6]. Both include the proportional distribution of fishing across SSMUs (“Annual Distribution”), and how this proportion was distributed seasonally within each SSMU (“Seasonal Distribution”). Thus, annual distribution sums to one by column, seasonal distribution by row (“Summer” + “Winter”).
(DOCX)

S1 Fig Schematic of the decomposition process. An arbitrary modeled marine area (A) is shown divided in its original spatial units. Table B denotes the proportional information used to update appropriate parameters and state variables for revising the spatial units. Decomposition creates updated spatial units (C) for the modeled marine area. Labels in (C) denote the new spatial units, based on original names, percentage of foraging, and whether or not the unit is open to fishing.
(TIF)

S2 Fig Comparing output from the decomposed model with that of the original. Comparison of original model output (darker colors, solid lines) with that from a simple decomposed model (lighter colors, dashed lines) for abundance (A) and recruitment (B), using the parameterization of full krill movement as passive drifters and a linear relationship between krill predators and krill availability (note that both outputs completely overlap).
(TIF)

S3 Fig Outcomes for whales by SSMU in both MPA scenarios. Changes in whale abundance with an MPA relative to the No MPA reference (i.e. MPA/No MPA), with the top row (A and B) the Regional redistribution of displaced catch, the middle row (C, D) the Local alternative, and bottom row (E, F) the Current alternative. The left column illustrates results from the D1MPA scenario (A, C, E) and the right from US10 (B, D, F). Grey indicates areas where the species group is not modeled to recruit. Note that changes are relative to the no MPA scenario within each SSMU, not to overall change.
(TIF)

S4 Fig Outcomes of both MPA scenarios for fish by SSMU. Change in fish abundance with the MPA relative to the No MPA scenario (i.e. MPA/No MPA); all details as in S3 Fig.
(TIF)

S5 Fig Difference in catch and allocation under an MPA scenario by SSMU. (A) Catch in the final year of the No MPA model (x-axis) is compared with the final catch with an MPA (y-axis); (B) the relative difference between the MPA and No MPA (MPA/No MPA) is considered for the final catch against the allocation, i.e. the relative difference between an MPA scenario and the No MPA reference in catch allocation (x-axis) versus realized catch (y-axis). In both graphs, the MPA scenario is designated by marker shape (circles for D1MPA, triangles for US10), and the redistribution method is indicated by the color (Regional is in blue, Local in green, and Current in orange). The dashed grey line in (A) indicates that catch would be the same with or without an MPA, with points below where catch declined with the MPA, and points above where catch increased. In (B), this line indicates where the relative difference between the No MPA and MPA scenarios is the same for the initial allocation and the final realized catch, with points below meaning catch was lower than anticipated given the allocation, and points about denoting catch as higher than anticipated. SSMU 5 is indicated in both (A) and (B) with an asterisk (*).
(TIF)

S6 Fig Probability of a threshold violation with and without an MPA by SSMU. The probability of a threshold violation in the No MPA scenario (x-axis) is compared with that probability in an MPA scenario (y-axis). The MPA scenario is designated by marker shape (circles for D1MPA, triangles for US10), and redistribution method is indicated by color (Regional in blue, Local in green, and Current in orange). The dashed grey line indicates where the probability would be the same with or without an MPA. Points above indicate where the probability of a threshold violation is higher with an MPA. Groups of points (i.e. for both MPA scenarios and all redistribution alternatives) denoting offshore SSMUs are circled in black.
(TIF)

S1 Dataset Update energy parameter, *p*_*k*,*i*←*j*,*s*_, in the ecosystem model. Values for the proportion of krill-derived energy that predators breeding in SSMU *i* obtain from SSMU *j*, *p*_*k*,*i*__←_. _*j*,*s*_, decomposed to the open and closed areas of the original SSMU.
(XLSX)

S2 Dataset Update to instantaneous rate of krill movement. Values for the parameter *v*_*i→j*,*s*_, instantaneous rates of krill movement from area *i* to area *j* in the ecosystem model, decomposed from original SSMUs to the open and closed areas within each SSMU.
(XLSX)
