# Supplementary material for: What’s the catch? Profiling the benefits and costs associated with marine protected areas and displaced fishing in the Scotia Sea
Source: PLoS One. 2020 Aug 12;15(8):e0237425. doi: 10.1371/journal.pone.0237425 (PMC7423141; doi:10.1371/journal.pone.0237425)
Supplement: S1 File — (DOCX) [file pone.0237425.s001.docx]

What’s the catch? Profiling the benefits and costs associated with marine protected areas and displaced fishing in the Scotia Sea

**Short title: Marine protected areas and displaced fishing in the Scotia Sea**

Emily S. Klein^1,2*^ and George M. Watters^1^

### The decomposition process

The ecosystem model we employed here [1] has previously been used at the spatial scale of small-scale management units (SSMUs, Fig 1 in the main text), yet emerging management needs, namely the evaluation of marine protected areas (MPAs), require insights at finer spatial scales. To address these needs, we developed code in the R programming language [2] to modify the model for implementation at finer spatial scales. The application of this code decomposed specific model input at the original SSMU scale into two portions, one outside the MPA and open to fishing, and one inside the MPA and closed to fishing.

In order to decompose an SSMU-scale parameterization of the model using the new code, several aspects must be defined: (1) proportional areas inside and outside the MPA relative to the original SSMU, (2) distributions of predator foraging between the two areas, (3) whether an area was open or closed to fishing, and (4) the distribution of fishing displaced from the closed areas across open areas according to the redistribution alternatives (see main text). These aspects were then used to decompose the original areas into smaller units (Fig S1). We used these aspects to update scale-dependent parameter and state variable input to the model (Tables S1-S3), assuming all other aspects remained unchanged. Then we ran the ecosystem model decomposed into the open and closed areas to evaluate the MPA scenarios. However, comparing our results with previous and future uses of the ecosystem model and reporting results at spatial scales relevant to management required summarizing our outputs at the SSMU scale.

We delineated most predator groups (penguins, seals, and fish) as reproducing and recruiting within the areas closed to fishing. For this reason, some parameters and state variables, such as maximum recruitment (*Rpeak_k,i,s_*, Table S2), did not need to be delineated by the open and closed portions of the original SSMU because the populations themselves were not divided. We found this approach appropriate given the closed portions were generally coastal where most predators occur (e.g., all penguins breed on land along the coast, see Fig 1 in main text). However, as in Watters et al. [1], we modeled whales as only recruiting to the pelagic areas, which were also those open to fishing.

Based on available telemetry data, predator groups were allowed to forage across all areas in the model, both within and outside the MPA. We modified season-specific distributions of foraging by penguins, whales, and seals using recent analyses of foraging location data for these groups [e.g. 3]. Briefly, a state-space model [4] was fitted to the data from each deployment and positions were interpolated on an hourly time step. Next, 50 alternative track lines for each deployment were simulated. The simulated tracks account for uncertainty in the location estimates and provide distributions of positions from which the proportion of time spent inside or outside the closed areas defined by each MPA scenario could be estimated. Resulting updated foraging is provided in data files accompanying this manuscript.

Because data is lacking (e.g. there is no fish tracking data), we assumed that fish forage, krill recruit and occur, and the fishery catches krill randomly within each SSMU, and allocated their distributions to portions of the SSMU inside and outside the MPA based on the MPA area relative to that of the original SSMU. For our No MPA reference, fishing was allowed in the MPA portions, but was not implemented there in scenarios with an MPA in place. Effort was conserved, and displaced fishing was redistributed based on three redistribution alternatives (main text). We recognize that these assumptions are simplistic: fish, krill, and the fishery have patchy spatial distributions at scales finer than the SSMU scale. More detailed characterizations of these distributions are topics of ongoing and future work.

We note that one parameter, maximum per-capita potential consumption (*Q^*^*), was included in our updates to predator foraging due to new information. We incorporate this update in data files on foraging updates accompanying this text. Another parameter, adult krill abundance that produces half of maximum recruitment, *β_i_*, was not updated. Instead, it was set at the same low level in Watters et al. [1] for both MPA and non-MPA portions of an SSMU. As in Watters et al. [1], this maintained a stock-recruitment relationship for krill that varied around a mean, as additional information defining a more sophisticated stock-recruitment relationship for krill is not available at this time.

Krill fishery catch was implemented based on the spatial and seasonal proportions of catch, 2009-2017 [5]. The proportions used here is compared with previous implementations of the model in Table S4.

Finally, we note one additional parameter, maximum per-capita potential consumption (*Q*_k,j,s_*), was updated given new information for a few species in specific locations (S2 Table). For whales in winter, it was updated to 48705863.62 in SSMU 1 and 53583388 in SSMU 9 (the only two SSMUs where whales are modeled to recruit). For seals in winter, it was updated to 1960605.762 in SSMUs 3, 4, and 7.

Following these updates, we checked that outcomes from the decomposed model did not alter model dynamics (Fig S2).

### Results for additional predator groups

We assessed MPA outcomes for all krill-dependent predator groups in our ecosystem model. Additional figures for the whale and fish groups are provided here (Fig S3 and S4).

### Further assessment of fishery outcomes

We also provide additional fishery outcomes in the MPA scenarios (Fig S5 and S6). As indicated in the main text, Fig S5A identifies declines in catch in SSMU 5 (designated by an asterisk) as differentiated from all other areas, which generally experienced increases or no change in catch. However, the redistribution alternatives mean catch allocation differs between the No MPA reference and the MPA scenarios (i.e. catch displaced from a closed area is redistributed based on the alternative, thus changing the allocation in the model). To assess whether changes in catch simply reflect this modeling choice, we compared the relative allocation (MPA/No MPA) against relative catch (MPA/No MPA) (Fig S5B). If the difference in catch with an MPA was explained by the difference in allocation, points in Fig S5B would lie along the 1:1 line. Instead, many points lie above this line, indicating that relative catch was higher in those areas than would be expected given the difference in allocation. Fig S5B also shows that the relative difference in catch for SSMU 5 can, at least in part, be explained by allocation via the Regional and Current alternative, although catch there was less with the Local alternative.

We also include relative differences in threshold violations by SSMU and redistribution alternative, comparing the MPA scenarios and the No MPA reference (Fig S6). This shows greater probability of a violation with an MPA across all SSMUs––no SSMU had a lower probability of a violation with an MPA. This is in part to be expected, given displacement by closed areas mean increased effort in smaller areas of an SSMU. We also note that the offshore SSMUs (groups circled in the figure) have among the highest probability of a threshold violation (ca. 0.4 to >0.6), but, for these areas, this is not demonstrably changed by fishing displaced there from either MPA scenario.

### References

1. Watters GM, Hill SL, Hinke JT, Matthews J, Reid K. Decision-making for ecosystem-based management: evaluating options for a krill fishery with an ecosystem dynamics model. Ecol App. 2013;23(4): 710-725. doi: 10.1890/12-1371.1.
2. R Core Team. R: A language and environment for statistical computing. R Foundation for Statistical Computing. Vienna, Austria; 2017. http://www.R-project.org/.
3. Hinke JT, Cossio AM, Goebel ME, Reiss CS, Trivelpiece WZ, Watters GM. Identifying risk: Concurrent overlap of the Antarctic krill fishery with krill-dependent predators in the Scotia Sea. PLOS ONE. 2017; 12(1): e0170132. doi: 10.1371/journal. pone.0170132.
4. Johnson D, London J, Lea M-A, Durban J. Continuous-time correlated random walk model for animal telemetry data. Ecology. 2008; 89: 1208–1215.
5. Commission for the Conservation of Antarctic Marine Living Resources (CCAMLR). Statistical Bulletin. 2018. Report No.: 29. Available from <https://www.ccamlr.org/en/meetings/26>.
