# Supplementary material for: What’s the catch? Profiling the benefits and costs associated with marine protected areas and displaced fishing in the Scotia Sea
Source: PLoS One. 2020 Aug 12;15(8):e0237425. doi: 10.1371/journal.pone.0237425 (PMC7423141; doi:10.1371/journal.pone.0237425)
Supplement: S1 Table — Spatial unit areas (m2) used in the original SSMU ecosystem model, with areas inside and outside the MPA calculated in the decomposed model for the D1MPA and US10 scenarios. (DOCX) [file pone.0237425.s002.docx]

**S1 Table. Update of area parameter, *A_i_*, in the ecosystem model**.

|  | Original areas (km^2^) | Areas used in the decomposed model (km^2^) | | | |
| --- | --- | --- | --- | --- | --- |
|  | Original SSMU (*A_i_*) | **D1MPA (*A_i_*)** | | **US 10 (*A_i_*)** | |
| SSMU |  | *Outside MPA* | *Inside MPA* | *Outside MPA* | *Inside MPA* |
| 1 | 422,000,000 | 407,778,600 | 14,221,400 | 361,527,400 | 60,472,600 |
| 2 | 35,060,000 | 24,300,086 | 10,759,914 | 13,792,604 | 21,267,396 |
| 3 | 15,068,000 | 7,491,810 | 7,576,190 | 8,460,682 | 6,607,318 |
| 4 | 15,584,000 | 10,358,685 | 5,225,315 | 6,375,414 | 9,208,586 |
| 5 | 21,017,000 | 7,412,696 | 13,604,304 | 5,189,097 | 15,827,903 |
| 6 | 27,447,000 | 15,919,260 | 11,527,740 | 21,312,596 | 6,134,405 |
| 7 | 35,322,000 | 22,969,897 | 12,352,103 | 35,322,000 | 0 |
| 8 | 58,704,000 | 48,730,190 | 9,973,810 | 58,222,281 | 481,719 |
| 9 | 809,000,000 | 715,722,300 | 93,277,700 | 715,722,300 | 93,277,700 |
| 19 | 15,569,000 | 13,853,296 | 1,715,704 | 15,569,000 | 0 |
| 11 | 10,251,000 | 7,009,634 | 3,241,366 | 10,251,000 | 0 |
| 12 | 14,954,000 | 10,644,257 | 4,309,743 | 14,954,000 | 0 |
| 13 | 920,000,000 | 920,000,000 | 0 | 920,000,000 | 0 |
| 14 | 42,119,000 | 42,119,000 | 0 | 42,119,000 | 0 |
| 15 | 53,735,000 | 53,735,000 | 0 | 53,735,000 | 0 |

Spatial unit areas (m^2^) used in the original SSMU ecosystem model, with areas inside and outside the MPA calculated in the decomposed model for the D1MPA and US10 scenarios. Zeros in the “*Inside MPA*” column denote there is not MPA (and therefore no closed area) in these SSMUs.
