# Supplementary material for: What’s the catch? Profiling the benefits and costs associated with marine protected areas and displaced fishing in the Scotia Sea
Source: PLoS One. 2020 Aug 12;15(8):e0237425. doi: 10.1371/journal.pone.0237425 (PMC7423141; doi:10.1371/journal.pone.0237425)
Supplement: S2 Table — Description of the parameters in our ecosystem model and whether the parameter or variable was adjusted in the decomposition process (white rows) (adopted from Tables B2 and B3, Watters et al. 2013). (DOCX) [file pone.0237425.s003.docx]

**S2 Table**. **Model parameters and state variables**.

| **Parameter or Variable** | **Description** | **Decomposed?** | **Process for decomposition** | ***Reference or data file*** |
| --- | --- | --- | --- | --- |
| *Predators* | | | | |
| $\bar{M}_{k,i,s}$ | Mean instantaneous rate of natural mortality | No | N/A | Watters et al. 2013 |
| $\upsilon_{k,i,s}$ | Level of foraging performance that distinguishes a good year from a bad year | No | N/A | Watters et al. 2013 |
| $z_{k,i,s}$ | Proportion of potential variation in survival that is explained by variations in mean per-capita foraging performance | No | N/A | Watters et al. 2013 |
| $\rho'_{k,i}$ | Age at recruitment to adult stage | No | N/A | Watters et al. 2013 |
| $\alpha'_{k,i,s}$ | Maximum per-capita recruitment at low adult abundance when all adults breed | No | N/A | Watters et al. 2013 |
| $Rpeak_{k,i,s}$ | Maximum recruitment when all adults breed | No | N/A | Watters et al. 2013 |
| $Speak_{k,i,s}$ | Adult abundance that produces maximum recruitment | No | N/A | Watters et al. 2013 |
| $\varphi'_{k,i,s}$ | Shape parameter determining ratio of effective breeder abundance to adult abundance | No | N/A | Watters et al. 2013 |
| $Q_{k,i,s}^{*}$ | Maximum per-capita potential consumption | No, but updated with new data for some species group | N/A | Watters et al. 2013, updates in Supporting Information |
| *Jphi* | Shape parameter that scales potential recruitment based on mean per-capita foraging success during first winter of life | No | N/A | Watters et al. 2013 |
| $\kappa_{k,i,s}$ | Krill density at which predators achieve half of maximum potential per-capita consumption | No | N/A | Watters et al. 2013 |
| $q_{k,i,s}$ | Functional response shape parameter | No | N/A | Watters et al. 2013 |
| $p_{k,i\leftarrow j,s}$ | Proportion of krill-derived energy that predators breeding in SSMU *i* obtain from SSMU *j* | Yes | Proportions of energy updated for predators and distributed among the open and closed portions of an SSMU using new tracking data [3] | S1 Dataset |
| $c_{k,i}$ | Competitive strength of predators relative to that of the fishery | No | N/A | Watters et al. 2013 |
| $\Omega_{k,i,0}$ | Initial potential demand for krill by a single predator stock breeding in SSMU *i* | No | N/A | Watters et al. 2013 |
| $P_{k,i,0}$ | Initial abundance of predators that breed in SSMU *i* | Updated, but  not decom-posed into open & closed areas | Abundance of predator group in SSMU *i* modeled in the MPA area of SSMU *i*; no abundance in non-MPA area of SSMU *i* | S3 Table |
| init.type | Specifies whether initial abundance is input directly or determined from consumption | No | N/A | Watters et al. 2013 |
| *Krill* | | | | |
| *$v_{i\to j,s}$* | Instantaneous rate of krill movement from area *i* to area *j;* rates are set as a proportion of the overall krill that move from SSMU *i* to SSMU *j* in season *s.* | Yes | The proportion of krill that move from *i* to *j* was assumed to be the same for the portions inside and outside an MPA as in the original SSMU (e.g. if 10% of krill from SSMU *i* move to *j*, then 10% of krill in the open area of SSMU *i* also move to *j*). This amount is then divided into the open and closed portions in SSMU *j* based on the proport-ional area of each MPA (i.e. proportions in S3 Table; e.g. if the non-MPA and MPA portions in *j* are 60% and 40% of the original area of *j*, then the 10% is further divided 60:40 to the portions in *j*). | S2 Dataset |
| $K_{i,t}$ | Abundance of krill | Yes, via initial density  (*$K_{i,0}/A_{i}$*) | See **below | S3 Table |
| *$M0_{i,s}$* | Instantaneous rate of natural, non-predation mortality | No | N/A | Watters et al. 2013 |
| *$\alpha_{i,s}$* | Maximum recruitment | Yes | Original parameter multiplied by the proportional area of the initial SSMU for the open and closed portions (i.e. proportions in S3 Table) | S3 Table |
| *$\beta_{i,s}$* | Adult abundance that produces half of maximum recruitment | No | N/A | Watters et al. 2013 |
| $\rho$ | Age at recruitment to adult stage | No | N/A | Watters et al. 2013 |
| $\varphi_{i,s}$ | Scalar that mediates environmental effects on krill recruitment | No | N/A | Watters et al. 2013 |
| $X_{t}$ | Environmental index influencing recruitment | No | N/A | Watters et al. 2013 |
| *$\sigma^{2}$* | Process variance in ln(recruitment) | No | N/A | Watters et al. 2013 |
| $\delta_{i}$ | Fraction of abundance available for harvest and predation | No | N/A | Watters et al. 2013 |
| *$K_{i,0}/A_{i}$* | Initial density of krill | Yes | Initial density of krill assumed to be constant across the SSMU, therefore this was multiplied by the proportional area of the original SSMU for each open and closed area (i.e. proportions in S3 Table) | S3 Table |
| *$\bar{w}_{i}$* | Average weight of an individual krill | No | N/A | Watters et al. 2013 |
| *Fishery* | | |  |  |
| $C_{H,i}$ | Historical catch of krill | Yes | Historical catch from inside the MPA portion of the SSMU is redistributed based on the redistribution options | S4 Table |
| $A_{i}$ | [Area of the SSMU]*[proportion of area inside and outside MPA] | Yes | Initial area of SSMU decomposed based on the proportion of the area of the SSMU that is inside or outside the MPA under each MPA scenario | S1 Table |
| $\gamma$ | Overall harvest rate | No | N/A | Watters et al. 2013 |
| $L$ | Precautionary catch limit | No | N/A | Watters et al. 2013 |
| $\eta_{i}$ | Threshold krill density (g ·m^-2^) that sets $C_{i,t}=F_{i,t}=0$ | No | N/A | Watters et al. 2013 |
| $f_{i,s}$ | Proportion of allocated catch taken in season *s* | No | N/A | Watters et al. 2013 |
| $c_{F,i}$ | Competitive strength of fishery relative to those of predators | No | N/A | Watters et al. 2013 |

Description of the parameters in our ecosystem model and whether the parameter or variable was adjusted in the decomposition process (white rows) (adopted from Tables B2 and B3, Watters et al. 2013).
