# Supplementary material for: What’s the catch? Profiling the benefits and costs associated with marine protected areas and displaced fishing in the Scotia Sea
Source: PLoS One. 2020 Aug 12;15(8):e0237425. doi: 10.1371/journal.pone.0237425 (PMC7423141; doi:10.1371/journal.pone.0237425)
Supplement: S3 Table — For krill, the initial density was multiplied by the proportion of area in either the MPA or no MPA portions: “inside MPA” and “outside MPA” columns sum to the “Original model” column. (DOCX) [file pone.0237425.s004.docx]

**S3 Table**. **Additional updated input for krill and krill-dependent predators in the decomposed model versus the original.**

|  |  | **D1 MPA** | | | | **US 10** | | | |
| --- | --- | --- | --- | --- | --- | --- | --- | --- | --- |
| SSMU | **Original Model** | Inside MPA | | Outside MPA | | Inside MPA | | Outside MPA | |
| ***Krill initial density (K_i,0_ /A_i_)*** | | | | | | | | | |
| 1 | 39.2 | 1.32104 | | 37.87896 | | 5.61736 | | 33.58264 | |
| 2 | 272 | 83.4768 | | 188.5232 | | 164.9952 | | 107.0048 | |
| 3 | 272 | 136.7616 | | 135.2384 | | 119.272 | | 152.728 | |
| 4 | 75.4 | 25.28162 | | 50.11838 | | 44.55386 | | 30.84614 | |
| 5 | 271.92 | 176.0138 | | 95.90618 | | 204.783 | | 67.13705 | |
| 6 | 271.92 | 114.2064 | | 157.7136 | | 60.77412 | | 211.1459 | |
| 7 | 271.92 | 95.09042 | | 176.8296 | | 271.92 *(no MPA in this SSMU)* | | | |
| 8 | 271.92 | 46.19921 | | 225.7208 | | 2.231346 | | 269.6887 | |
| 9 | 62.94 | 7.256982 | | 55.68302 | | 7.256982 | | 55.68302 | |
| 19 | 638.84 | 70.40017 | | 568.4398 | | 638.84 *(no MPA)* | | | |
| 11 | 638.84 | 202.0012 | | 436.8388 | | 638.84 *(no MPA)* | | | |
| 12 | 638.84 | 184.1137 | | 454.7263 | | 638.84 *(no MPA* | | | |
| 13 | 62.94 | 62.94 *(no MPA in this SSMU)* | | | | 62.94 *(no MPA)* | | | |
| 14 | 67.68 | 67.68 *(no MPA)* | | | | 67.68 *(no MPA)* | | | |
| 15 | 67.68 | 67.68 *(no MPA)* | | | | 67.68 *(no MPA)* | | | |
| ***Krill maximum recruitment (α_i,s_) – movement scenarios*** | | | | | | | | | |
| 1 | 1.73E+12 | | 2.48E+11 | | 1.48E+12 | | 2.48E+11 | | 1.48E+12 |
| 2 | 2.07E+13 | | 1.25E+13 | | 8.13E+12 | | 1.25E+13 | | 8.13E+12 |
| 3 | 1.45E+13 | | 6.35E+12 | | 8.13E+12 | | 6.35E+12 | | 8.13E+12 |
| 4 | 2.82E+12 | | 1.66E+12 | | 1.15E+12 | | 1.66E+12 | | 1.15E+12 |
| 5 | 1.86E+13 | | 1.40E+13 | | 4.6E+12 | | 1.4E+13 | | 4.6E+12 |
| 6 | 1.37E+13 | | 3.06E+12 | | 1.06E+13 | | 3.06E+12 | | 1.06E+13 |
| 7 | 1.45E+13 | | 5.08E+12 | | 9.45E+12 | | 1.45E+13 *(no MPA)* | | |
| 8 | 2.71E+13 | | 2.23E+11 | | 2.69E+13 | | 2.23E+11 | | 2.69E+13 |
| 9 | 8.97E+08 | | 1.03E+08 | | 7.93E+08 | | 1.03E+08 | | 7.93E+08 |
| 19 | 3.51E+13 | | 3.86E+12 | | 3.12E+13 | | 8.97E+08 *(no MPA)* | | |
| 11 | 2.30E+13 | | 7.27E+12 | | 1.57E+13 | | 3.51E+13 *(no MPA)* | | |
| 12 | 3.08E+13 | | 8.89E+12 | | 2.2E+13 | | 3.08E+13 *(no MPA* | | |
| 13 | 7.66E+09 | | 7.66E+09 *(no MPA)* | | | | 7.66E+09 *(no MPA)* | | |
| 14 | 4.50E+09 | | 4.50E+09*(no MPA)* | | | | 4.50E+09 *(no MPA)* | | |
| 15 | 2.10E+09 | | 2.10E+09 *(no MPA)* | | | | 2.10E+09 *(no MPA)* | | |
| ***Krill maximum recruitment (α_i,s_) – no movement scenarios*** | | | | | | | | | |
| 1 | 1.73E+13 | 2.48E+12 | | 1.48E+13 | | 2.48E+12 | | 1.4E+12 | |
| 2 | 3.57E+12 | 2.17E+12 | | 1.40E+12 | | 2.17E+12 | | 6.01E+11 | |
| 3 | 1.07E+12 | 4.69E+11 | | 6.01E+11 | | 4.69E+11 | | 6.59E+11 | |
| 4 | 1.61E+12 | 9.51E+11 | | 6.59E+11 | | 9.51E+11 | | 5.26E+11 | |
| 5 | 2.13E+12 | 1.60E+12 | | 5.26E+11 | | 1.6E+12 | | 1.77E+12 | |
| 6 | 2.28E+12 | 5.10E+11 | | 1.77E+12 | | 5.1E+11 | | 1.4E+12 | |
| 7 | 2.15E+12 | 7.52E+11 | | 1.40E+12 | | 2.15E+12 *(no MPA)* | | | |
| 8 | 7.24E+12 | 5.94E+10 | | 7.18E+12 | | 5.94E+10 | | 7.18E+12 | |
| 9 | 2.39E+13 | 2.76E+12 | | 2.11E+13 | | 2.76E+12 | | 2.11E+13 | |
| 19 | 6.52E+11 | 7.19E+10 | | 5.80E+11 | | 7.19E+10 *(no MPA)* | | | |
| 11 | 8.91E+11 | 2.82E+11 | | 6.09E+11 | | 2.82E+11 *(no MPA)* | | | |
| 12 | 2.61E+12 | 7.52E+11 | | 1.86E+12 | | 7.52E+11 *(no MPA* | | | |
| 13 | 3.06E+13 | 3.06E+13*(no MPA)* | | | | 3.06E+13*(no MPA)* | | | |
| 14 | 6.00E+12 | 6.00E+12 *(no MPA)* | | | | 6.00E+12 *(no MPA)* | | | |
| 15 | 2.80E+12 | 2.80E+12 *(no MPA)* | | | | 2.80E+12 *(no MPA)* | | | |
| ***Penguin initial abundance (P_i,0_)*** | | | | | | | | | |
| 1 | 0 | 0 | | 0 | | 0 | | 0 | |
| 2 | 241,540 | 241,540 | | 0 | | 241,540 | | 0 | |
| 3 | 71,164 | 71,164 | | 0 | | 71,164 | | 0 | |
| 4 | 1,183,090 | 1,183,090 | | 0 | | 1,183,090 | | 0 | |
| 5 | 1,079,278 | 1,079,278 | | 0 | | 1,079,278 | | 0 | |
| 6 | 259,496 | 259,496 | | 0 | | 259,496 | | 0 | |
| 7 | 852,001 | 852,001 | | 0 | | 852,001 *(no MPA)* | | | |
| 8 | 715,053 | 715,053 | | 0 | | 715,053 | | 0 | |
| 9 | 0 | 0 | | 0 | | 0 | | 0 | |
| 10 | 1,812 | 1,812 | | 0 | | 1,812 *(no MPA)_* | | | |
| 11 | 463,307 | 463,307 | | 0 | | 463,307 *(no MPA)* | | | |
| 12 | 1,588,430 | 1,588,430 | | 0 | | 1,588,430 *(no MPA)* | | | |
| 13 | 0 | 0 (*no MPA*) | | | | 0 (*no MPA*) | | | |
| 14 | 6,642,811 | 6,642,811 *(no MPA*) | | | | 6,642,811 (*no MPA*) | | | |
| 15 | 564,496 | 564,496 *(no MPA)* | | | | 564,496 *(no MPA)* | | | |
| ***Seal initial abundance (P_i,0_)*** | | | | | | | | | |
| 1 | 0 | 0 | | 0 | | 0 | | 0 | |
| 2 | 0 | 0 | | 0 | | 0 | | 0 | |
| 3 | 413 | 413 | | 0 | | 413 | | 0 | |
| 4 | 7 | 7 | | 0 | | 7 | | 0 | |
| 5 | 0 | 0 | | 0 | | 0 | | 0 | |
| 6 | 0 | 0 | | 0 | | 0 | | 0 | |
| 7 | 34 | 34 | | 0 | | 34 *(no MPA)* | | | |
| 8 | 0 | 0 | | 0 | | 0 | | 0 | |
| 9 | 0 | 0 | | 0 | | 0 | | 0 | |
| 10 | 0 | 0 | | 0 | | 0 *(no MPA)* | | | |
| 11 | 0 | 0 | | 0 | | 0 *(no MPA)* | | | |
| 12 | 0 | 0 | | 0 | | 0 *(no MPA)* | | | |
| 13 | 0 | 0 (*no MPA*) | | | | 0 (*no MPA*) | | | |
| 14 | 70,208 | 70,208 *(no MPA)* | | | | 70,208 (*no MPA*) | | | |
| 15 | 700 | 700 *(no MPA)* | | | | 700 *(no MPA)* | | | |
| ***Whale initial abundance (P_i,0_)*** | | | | | | | | | |
| 1 | 2,680 | 0 | | 2,680 | | 0 | | 2,680 | |
| 2 | 0 | 0 | | 0 | | 0 | | 0 | |
| 3 | 0 | 0 | | 0 | | 0 | | 0 | |
| 4 | 0 | 0 | | 0 | | 0 | | 0 | |
| 5 | 0 | 0 | | 0 | | 0 | | 0 | |
| 6 | 0 | 0 | | 0 | | 0 | | 0 | |
| 7 | 0 | 0 | | 0 | | 0 *(no MPA)* | | | |
| 8 | 0 | 0 | | 0 | | 0 | | 0 | |
| 9 | 2,970 | 0 | | 2,970 | | 0 | | 2,970 | |
| 10 | 0 | 0 | | 0 | | 0 *(no MPA)* | | | |
| 11 | 0 | 0 | | 0 | | 0 *(no MPA)* | | | |
| 12 | 0 | 0 | | 0 | | 0 *(no MPA)* | | | |
| 13 | 0 | 0 (*no MPA*) | | | | 0 *(no MPA)* | | | |
| 14 | 0 | 0 *(no MPA)* | | | | 0 *(no MPA)* | | | |
| 15 | 0 | 0 *(no MPA)* | | | | 0 *(no MPA)* | | | |
| ***Fish initial abundance*** ***(P_i,0_)*** | | | | | | | | | |
| 1 | 8,402,727,132 | 8,402,727,132 | | 0 | | 8,402,727,132 | | 0 | |
| 2 | 834,429,245 | 834,429,245 | | 0 | | 834,429,245 | | 0 | |
| 3 | 301,932,508 | 301,932,508 | | 0 | | 301,932,508 | | 0 | |
| 4 | 322,045,302 | 322,045,302 | | 0 | | 322,045,302 | | 0 | |
| 5 | 438,862,551 | 438,862,551 | | 0 | | 438,862,551 | | 0 | |
| 6 | 574,691,426 | 574,691,426 | | 0 | | 574,691,426 | | 0 | |
| 7 | 679,683,642 | 679,683,642 | | 0 | | 679,683,642 *(no MPA)* | | | |
| 8 | 1,553,175,192 | 1,553,175,192 | | 0 | | 1,553,175,192 | | 0 | |
| 9 | 69,055,728,795 | 69,055,728,795 | | 0 | | 69,055,728,795 | | 0 | |
| 10 | 320,095,594 | 320,095,594 | | 0 | | 320,095,594 *(no MPA)* | | | |
| 11 | 196,909,670 | 196,909,670 | | 0 | | 196,909,670 *(no MPA)* | | | |
| 12 | 389,311,850 | 389,311,850 | | 0 | | 389,311,850 *(no MPA)* | | | |
| 13 | 248,777,387,143 | 248,777,387,143 *(no MPA)* | | | | 248,777,387,143 *(no MPA)* | | | |
| 14 | 1,140,779,048 | 1,140,779,048 *(no MPA)* | | | | 1,140,779,048 *(no MPA)* | | | |
| 15 | 1,477,796,628 | 1,477,796 ,628 *(no MPA)* | | | | 1,477,796 ,628 *(no MPA)* | | | |

For krill, the initial density was multiplied by the proportion of area in either the MPA or no MPA portions: “inside MPA” and “outside MPA” columns sum to the “Original model” column.
