# Supplementary material for: What’s the catch? Profiling the benefits and costs associated with marine protected areas and displaced fishing in the Scotia Sea
Source: PLoS One. 2020 Aug 12;15(8):e0237425. doi: 10.1371/journal.pone.0237425 (PMC7423141; doi:10.1371/journal.pone.0237425)
Supplement: S4 Table — Proportions used in earlier versions of the model are under “Previous Model” (Table S1 in [5]). The updates used here under “Current Model” are derived from catches taken during the 2009–2016 fishing seasons and under limits at finer spatial scales specified by current management [6]. Both include the proportional distribution of fishing across SSMUs (“Annual Distribution”), and how this proportion was distributed seasonally within each SSMU (“Seasonal Distribution”). Thus, annual distribution sums to one by column, seasonal distribution by row (“Summer” + “Winter”). (DOCX) [file pone.0237425.s005.docx]

**S4 Table**. **Proportional distributions of krill catch for each of the 15 SSMUs.**

| **SSMU** | **Previous model** | | | **Current model** | | |
| --- | --- | --- | --- | --- | --- | --- |
|  | Annual Distribution | Seasonal Distribution | | Annual Distribution | Seasonal Distribution | |
|  |  | *Summer* | *Winter* |  | *Summer* | *Winter* |
| 1 | 0.001 | 0.785 | 0.215 | 0.000^*^ | 0.984 | 0.016 |
| 2 | 0.030 | 0.442 | 0.558 | 0.076 | 0.406 | 0.594 |
| 3 | 0.022 | 0.447 | 0.553 | 0.040 | 0.418 | 0.582 |
| 4 | 0.013 | 0.759 | 0.241 | 0.026 | 0.801 | 0.199 |
| 5 | 0.120 | 0.302 | 0.699 | 0.250 | 0.313 | 0.687 |
| 6 | 0.060 | 0.371 | 0.629 | 0.148 | 0.395 | 0.605 |
| 7 | 0.004 | 0.835 | 0.165 | 0.006 | 0.998 | 0.002 |
| 8 | 0.001 | 0.732 | 0.268 | 0.000^*^ | 0.732^†^ | 0.268^†^ |
| 9 | 0.004 | 0.858 | 0.143 | 0.001 | 0.728 | 0.272 |
| 10 | 0.427 | 0.482 | 0.518 | 0.218 | 0.619 | 0.381 |
| 11 | 0.011 | 0.963 | 0.037 | 0.006 | 0.964 | 0.036 |
| 12 | 0.000 | 0.527 | 0.473 | 0.000 | 0.645 | 0.355 |
| 13 | 0.000 | 0.743 | 0.257 | 0.000 | 0.291 | 0.709 |
| 14 | 0.013 | 0.071 | 0.929 | 0.008 | 0.072 | 0.928 |
| 15 | 0.294 | 0.011 | 0.989 | 0.220 | 0.009 | 0.991 |

Proportions used in earlier versions of the model are under “Previous Model” (Table S1 in [1]). The updates used here under “Current Model” are derived from catches taken during the 2009-2017 fishing seasons and under limits at finer spatial scales specified by current management [5]. Both include the proportional distribution of fishing across SSMUs (“Annual Distribution”), and how this proportion was distributed seasonally within each SSMU (“Seasonal Distribution”). Thus, annual distribution sums to one by column, seasonal distribution by row (“Summer” + “Winter”).

^*^*Krill catch proportion in SSMU 1 is 0.000436, for SSMU 8 it is 0.*

^†^*In the 2017 CCAMLR Statistical Bulletin [46], catch of krill has been updated to report no catch in SSMU 8 for 2009-2017. Given that, in our model, we allow catch and redistribute catches displaced by the MPA there, we use earlier proportions [in 1] to update catch reallocations when needed for SSMU 8.*
