# Supplementary figures and images for: What’s the catch? Profiling the benefits and costs associated with marine protected areas and displaced fishing in the Scotia Sea
Source: PLoS One. 2020 Aug 12;15(8):e0237425. doi: 10.1371/journal.pone.0237425 (PMC7423141; doi:10.1371/journal.pone.0237425)

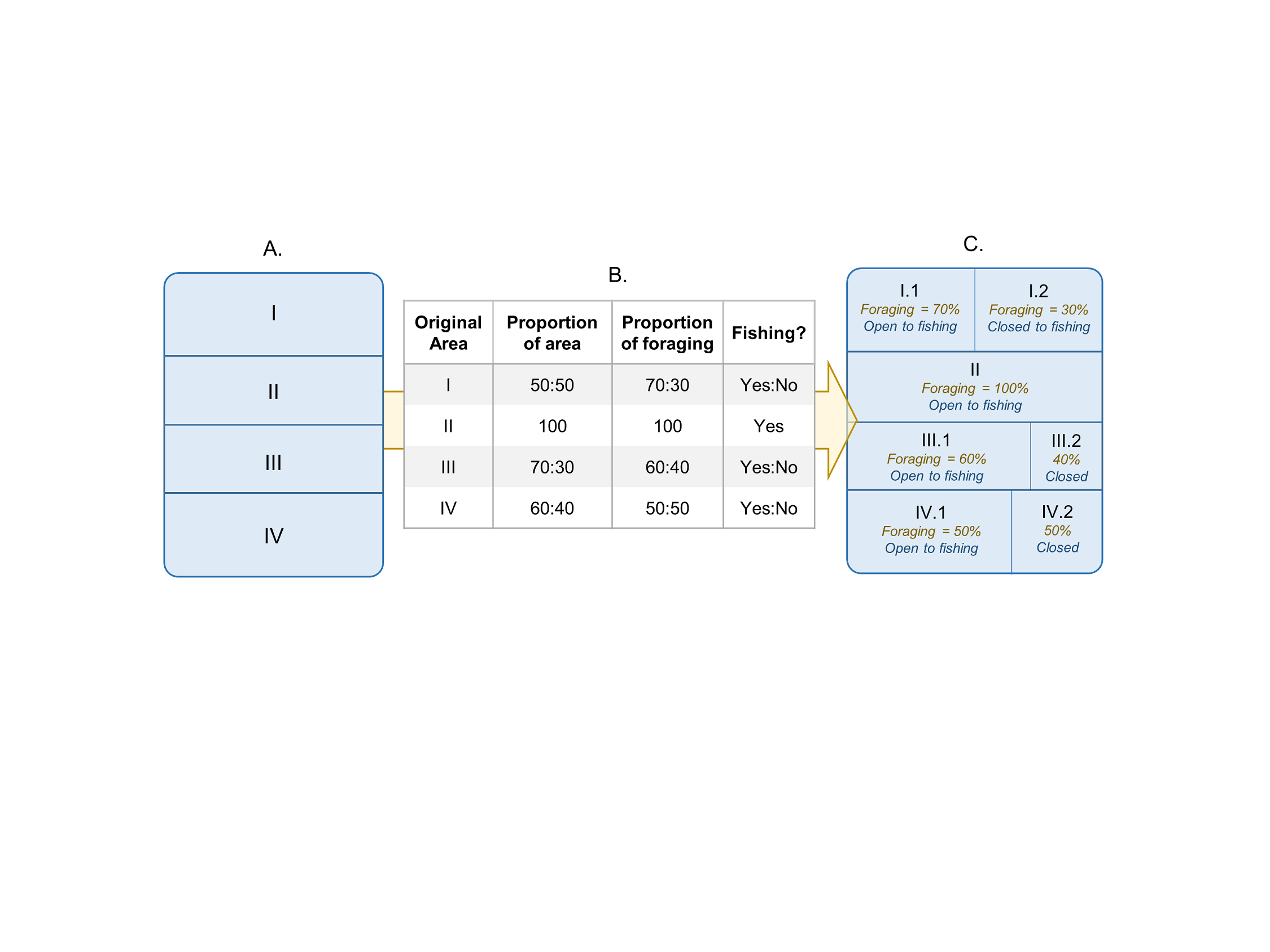

Supplement: S1 Fig — An arbitrary modeled marine area (A) is shown divided in its original spatial units. Table B denotes the proportional information used to update appropriate parameters and state variables for revising the spatial units. Decomposition creates updated spatial units (C) for the modeled marine area. Labels in (C) denote the new spatial units, based on original names, percentage of foraging, and whether or not the unit is open to fishing. (TIF) [file pone.0237425.s006.TIF]

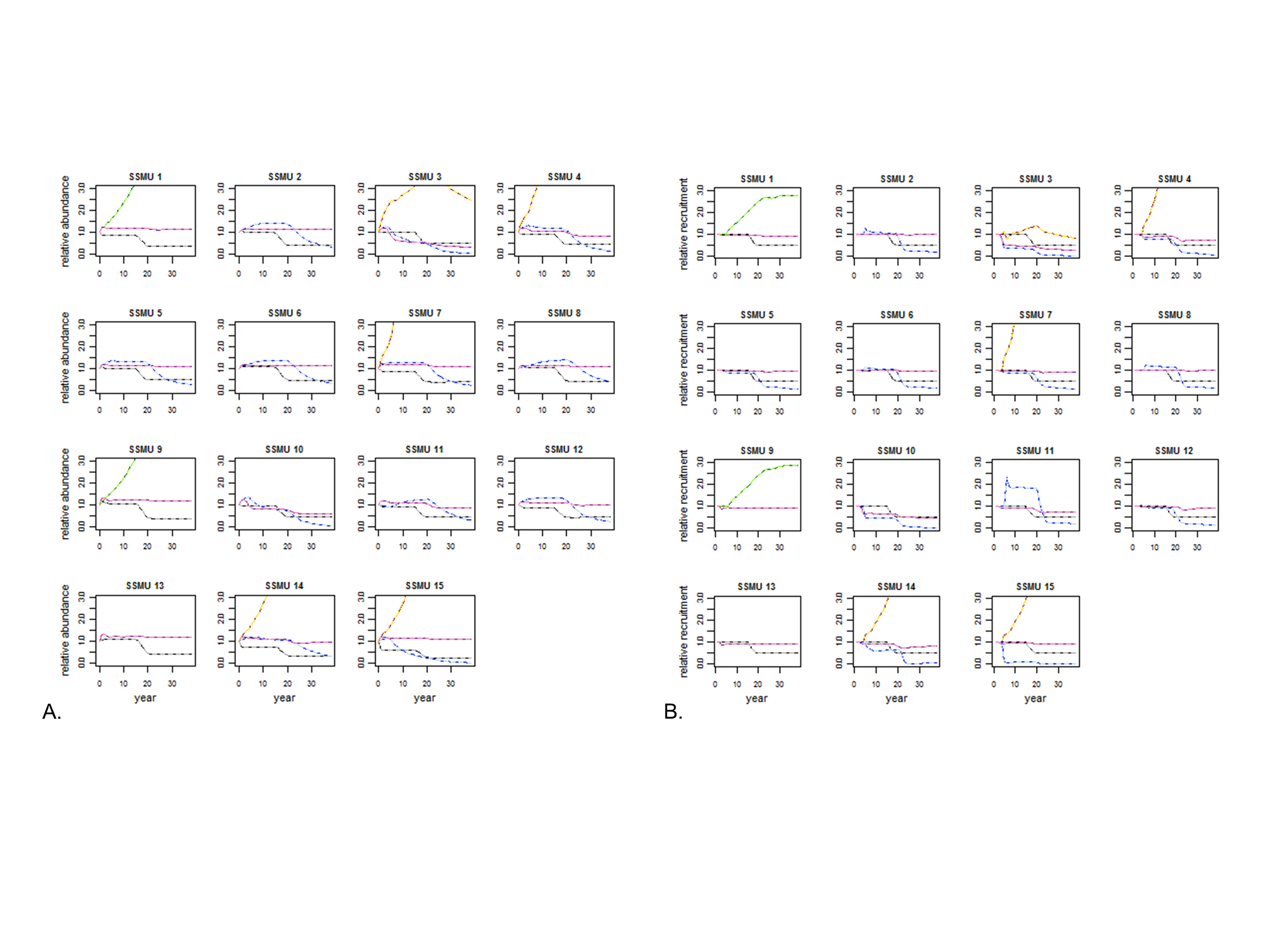

Supplement: S2 Fig — Comparison of original model output (darker colors, solid lines) with that from a simple decomposed model (lighter colors, dashed lines) for abundance (A) and recruitment (B), using the parameterization of full krill movement as passive drifters and a linear relationship between krill predators and krill availability (note that both outputs completely overlap). (TIF) [file pone.0237425.s007.TIF]

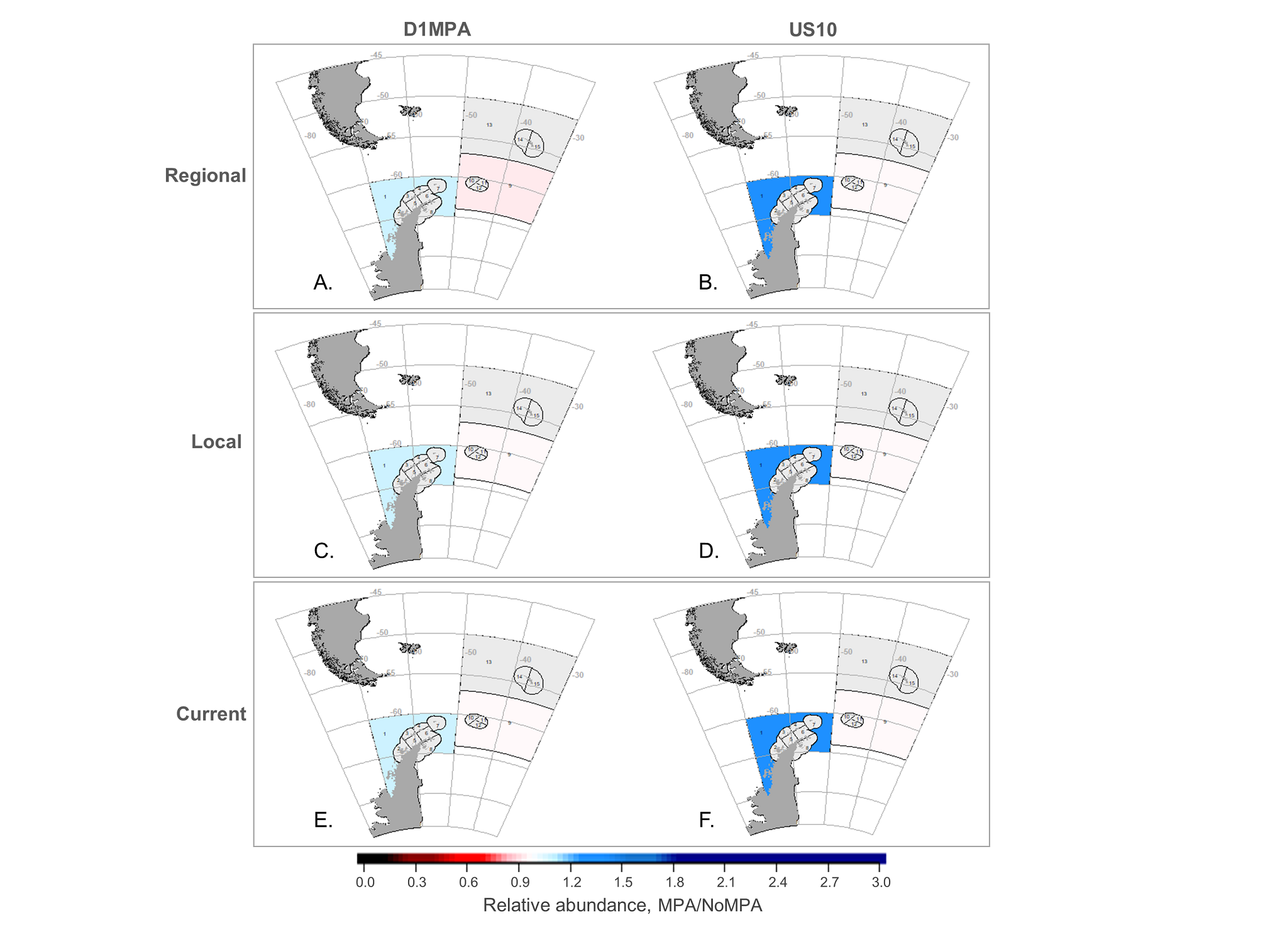

Supplement: S3 Fig — Changes in whale abundance with an MPA relative to the No MPA reference (i.e. MPA/No MPA), with the top row (A and B) the Regional redistribution of displaced catch, the middle row (C, D) the Local alternative, and bottom row (E, F) the Current alternative. The left column illustrates results from the D1MPA scenario (A, C, E) and the right from US10 (B, D, F). Grey indicates areas where the species group is not modeled to recruit. Note that changes are relative to the no MPA scenario within each SSMU, not to overall change. (TIF) [file pone.0237425.s008.TIF]

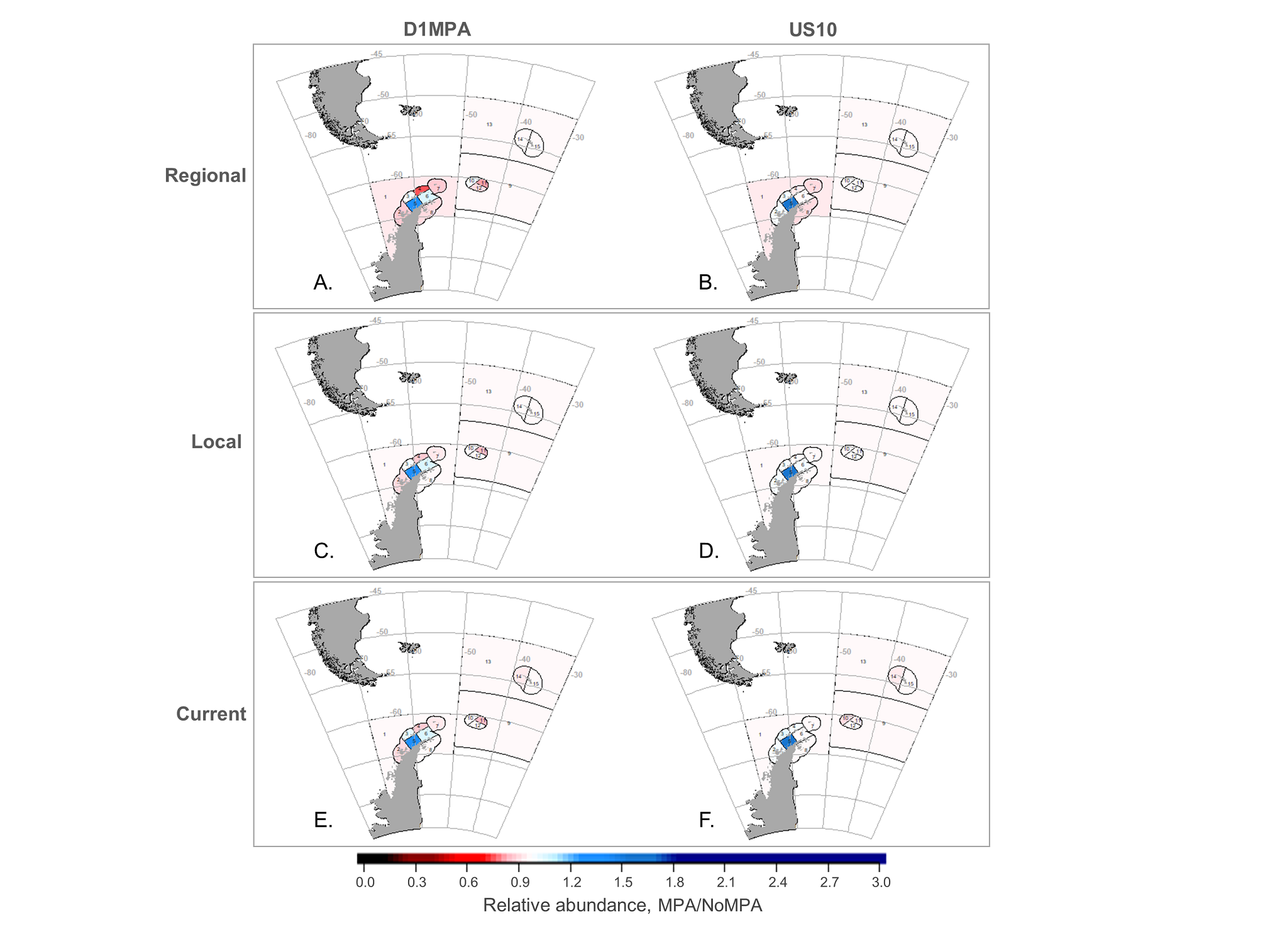

Supplement: S4 Fig — Change in fish abundance with the MPA relative to the No MPA scenario (i.e. MPA/No MPA); all details as in S3 Fig. (TIF) [file pone.0237425.s009.TIF]

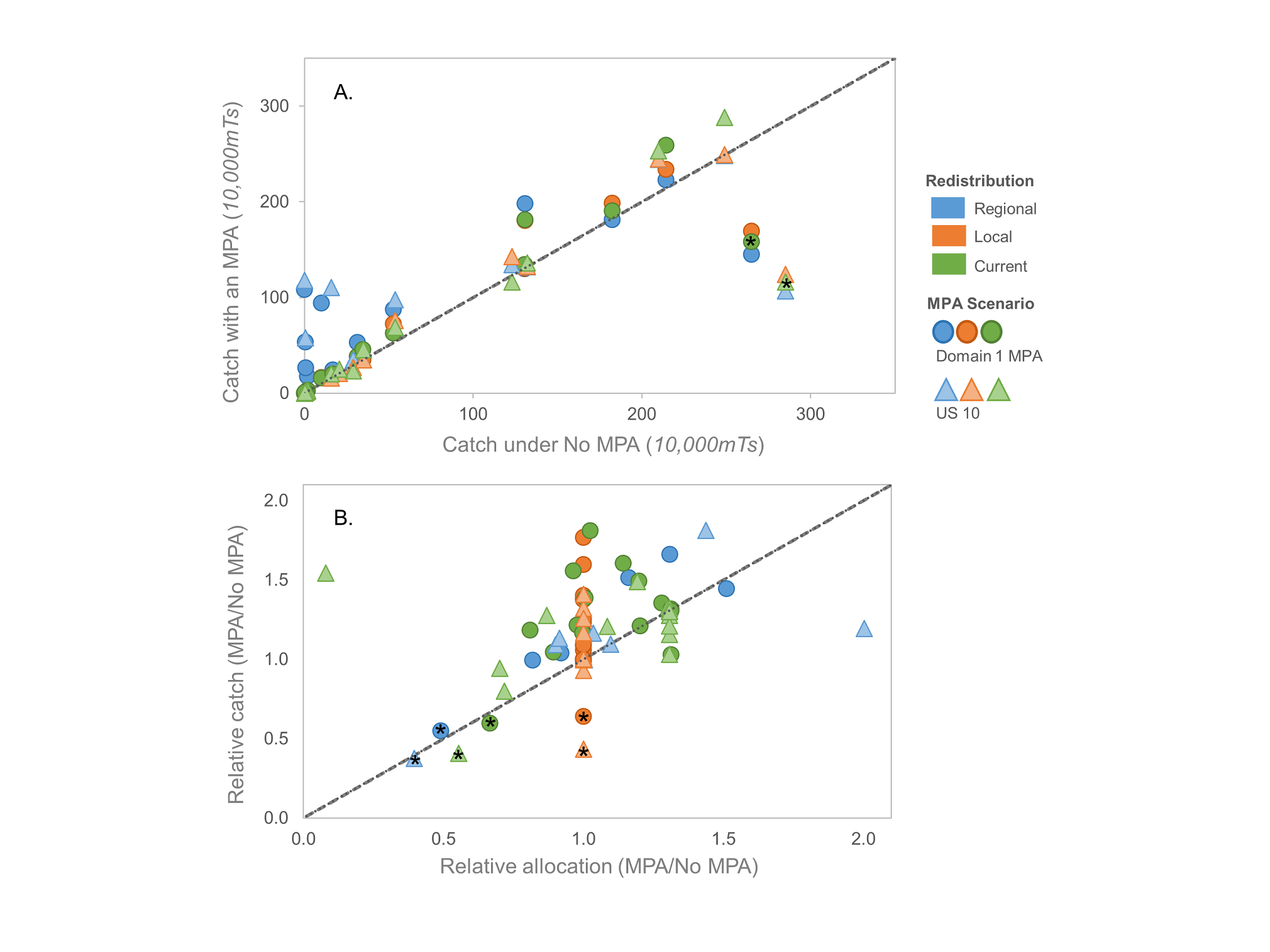

Supplement: S5 Fig — (A) Catch in the final year of the No MPA model (x-axis) is compared with the final catch with an MPA (y-axis); (B) the relative difference between the MPA and No MPA (MPA/No MPA) is considered for the final catch against the allocation, i.e. the relative difference between an MPA scenario and the No MPA reference in catch allocation (x-axis) versus realized catch (y-axis). In both graphs, the MPA scenario is designated by marker shape (circles for D1MPA, triangles for US10), and the redistribution method is indicated by the color (Regional is in blue, Local in green, and Current in orange). The dashed grey line in (A) indicates that catch would be the same with or without an MPA, with points below where catch declined with the MPA, and points above where catch increased. In (B), this line indicates where the relative difference between the No MPA and MPA scenarios is the same for the initial allocation and the final realized catch, with points below meaning catch was lower than anticipated given the allocation, and points about denoting catch as higher than anticipated. SSMU 5 is indicated in both (A) and (B) with an asterisk (*). (TIF) [file pone.0237425.s010.TIF]

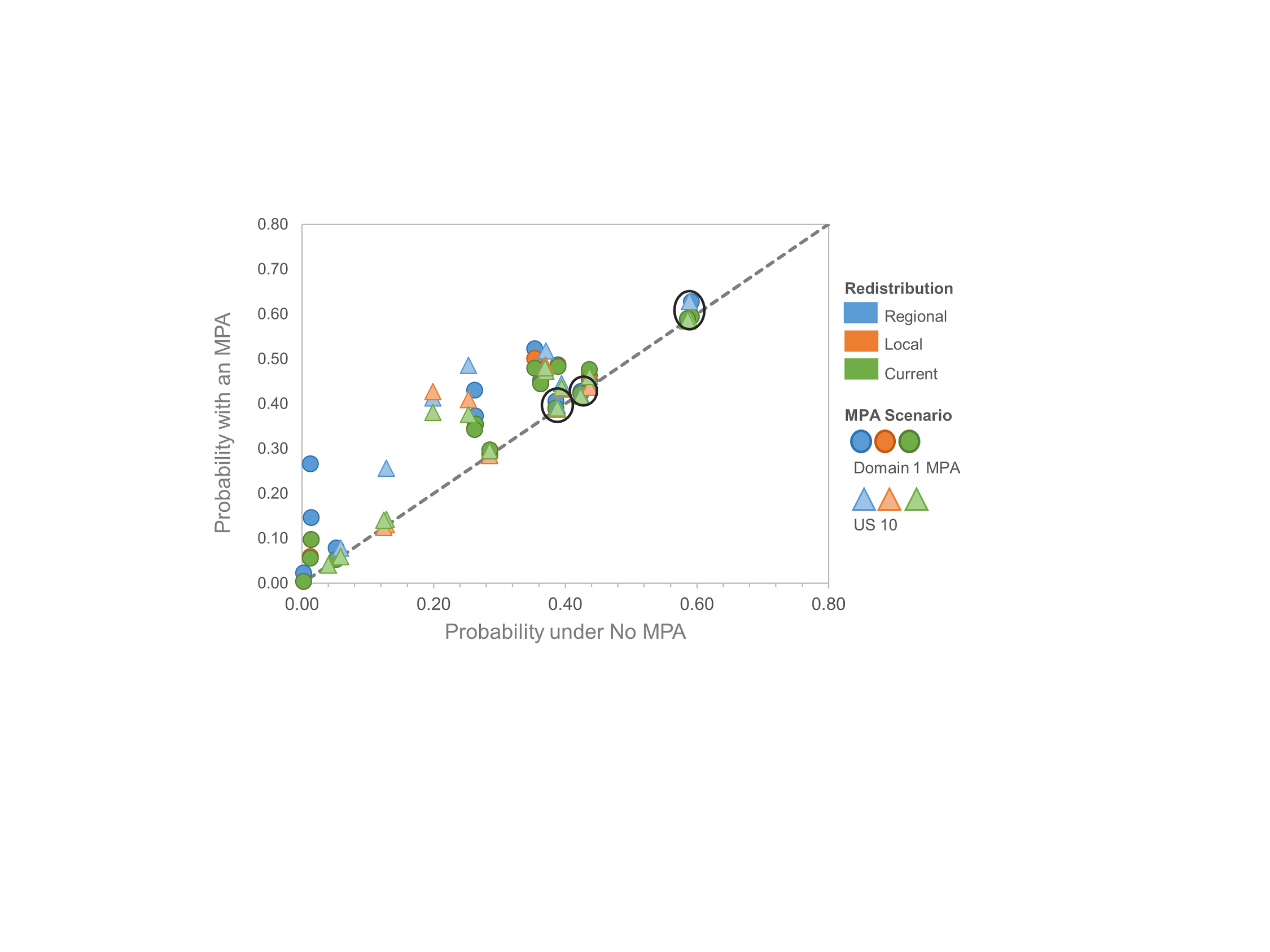

Supplement: S6 Fig — The probability of a threshold violation in the No MPA scenario (x-axis) is compared with that probability in an MPA scenario (y-axis). The MPA scenario is designated by marker shape (circles for D1MPA, triangles for US10), and redistribution method is indicated by color (Regional in blue, Local in green, and Current in orange). The dashed grey line indicates where the probability would be the same with or without an MPA. Points above indicate where the probability of a threshold violation is higher with an MPA. Groups of points (i.e. for both MPA scenarios and all redistribution alternatives) denoting offshore SSMUs are circled in black. (TIF) [file pone.0237425.s011.TIF]
